# Discovery and structure of a widespread bacterial ABC transporter specific for ergothioneine

**DOI:** 10.1038/s41467-022-35277-3

**Published:** 2022-12-08

**Authors:** Yifan Zhang, Giovanni Gonzalez-Gutierrez, Katherine A. Legg, Brenna J. C. Walsh, Cristian M. Pis Diez, Katherine A. Edmonds, David P. Giedroc

**Affiliations:** 1 Department of Chemistry, Indiana University, Bloomington, IN 47405-7102 USA; 2 Department of Molecular and Cellular Biochemistry, Indiana University, Bloomington, IN USA; 3 Fundación Instituto Leloir, Av. Patricias Argentinas 435, Buenos Aires, C1405BWE Argentina; 4 Present Address: Research and Exploratory Development Department, The Johns Hopkins University Applied Physics Laboratory, 11100 Johns Hopkins Road, Laurel, MD 20723 USA; 5 Present Address: Department of Chemistry, New York University, 100 Washington Square E, New York, NY 10003 USA

**Keywords:** Biophysical chemistry, Proteins

## Abstract

*L*-Ergothioneine (ET), the 2-thioimidazole derivative of trimethylhistidine, is biosynthesized by select fungi and bacteria, notably *Mycobacterium tuberculosis*, and functions as a scavenger of reactive oxygen species. The extent to which ET broadly functions in bacterial cells unable to synthesize it is unknown. Here we show that *spd_1642-1643* in *Streptococcus pneumoniae*, a Gram-positive respiratory pathogen, encodes an ET uptake ATP-binding cassette (ABC) transporter, designated EgtU. The solute binding domain (SBD) of EgtU, EgtUC, binds ET with high affinity and exquisite specificity in a cleft between the two subdomains, with cation-π interactions engaging the betaine moiety and a network of water molecules that surround the thioimidazole ring. EgtU is highly conserved among known quaternary amine compound-specific transporters and widely distributed in Firmicutes, including the human pathogens *Listeria monocytogenes*, as BilEB, *Enterococcus faecalis* and *Staphylococcus aureus*. ET increases the chemical diversity of the low molecular weight thiol pool in Gram-positive human pathogens and may contribute to antioxidant defenses in the infected host.

## Abstract (web summary)

The extent to which human bacterial pathogens broadly utilize ergothioneine as an antioxidant is unknown. Here, authors describe the discovery of a specific transporter for ergothioneine in a respiratory pathogen that is highly conserved among Firmicutes.

## Introduction

Cell-abundant low molecular weight (LMW) thiols maintain the reducing environment of the cytoplasm of bacterial cells and the cytosol of eukaryotic cells, and include the ubiquitous tripeptide glutathione (GSH)^1^. Bacteria unable to access glutathione often synthesize other thiols, including bacillithiol and mycothiol found in some Firmicutes and Actinomycetes, respectively^2,3^. These cell-abundant LMW thiols provide protection against endogenous or exogenous reactive oxygen and nitrogen species (ROS, RNS) as ROS scavengers to create thiol disulfides which are subsequently reduced, regenerating the free thiol and thus maintaining redox balance^4^. Bacterial LMW thiols are known to play key roles in oxidative and reductive stress responses in the infected host^5^.

Ergothioneine (ET) is a LMW thiol and trimethylamine (betaine) derivative of histidine with sulfur installed at the imidazole C2 position^6^. Unlike other LMW thiols that function as cellular redox buffers, ET is found in the thione tautomer rather than the thiol tautomer at physiological pH^7^ (Supplementary Fig. 1). This thiol-thione tautomerization significantly increases the thiol-disulfide reduction potential of ET relative to other LMW thiols thus endowing ET with properties of a highly effective scavenger of myriad ROS^8,9^. ET also chelates transition metals including Cu^+^, Cu^2+^, and Fe^2+^ and thus may impact metal and metal oxidation state speciation in cells^10–12^.

ET biosynthesis has been reported to occur only in select filamentous fungi including *Neurospora crassa*^13^, certain cyanobacteria^14^, Methylobacterium spp^15^., Burkholderia spp^16^., and in Actinomycetes, including the causative agent of tuberculosis, *Mycobacterium tuberculosis*, and its soil saprophyte, *Mycobacterium smegmatis*^17–19^. There have been no reports of ET biosynthesis in plants or animals^8,20^. In humans, ET is obtained from the diet and accumulates in tissues via an ergothioneine-specific transporter ETT, previously named organic cation/carnitine transporter I (OCTN1; SLC22A4)^21–23^, and a member of the Major Facilitator Superfamily (MFS). The expression level of ETT in various tissues has been used as proxy for the abundance and distribution of ET in animals^20,21^. ETT expression is high in the small intestine and the kidney which reflects dietary ET uptake and ET recovery from the urine, respectively^23,24^. High ETT expression has also been identified in blood cells, including erythrocytes in bone marrow, granulocytes, monocytes, and neutrophils, while detectable expression occurs in other tissues including the lung^23,25–27^. These studies suggest that ET is bioavailable in vertebrates and could be exploited by both resident commensals and pathogenic organisms to provide protection against host oxidative stressors; however, no widespread bacterial transporter for ET is known^8,20,21^.

Here, we describe the discovery and structural characterization of a bacterial ET transporter belonging to the ATP-binding cassette (ABC) superfamily. ABC transporters use the binding and hydrolysis of ATP to drive substrate translocation across the membrane, via two transmembrane domains (TMDs) and two cytoplasmic nucleotide-binding domains (NBDs). Prokaryotic ABC importers rely on high-affinity substrate-binding domains (SBDs) that dictate the specificity of the transporter. In Gram-negative bacteria, SBDs are typically soluble periplasmic proteins, while in Gram-positive bacteria, they are either anchored to the membrane by a covalently attached lipid or fused to the TMD as a single chain. These SBDs contain two structurally conserved subdomains, connected by a hinge region, with substrate binding in a cleft between the subdomains stabilizing a closed conformation that may be required for docking onto TMDs and delivery of substrate to the translocation channel^28^. Bacterial ABC importers can be subdivided into two classes, type I and type II according to the topology of their TMDs, and the two types appear to function by distinct mechanisms.

We show here that *spd_1642-1643* in the Gram-positive commensal and respiratory pathogen *Streptococcus pneumoniae* encodes a type II ABC transporter that is highly selective for ET. The cytoplasmic ATPase, denoted EgtUA, is encoded by *spd_1643*. The TMD (EgtUB) and SBD (EgtUC) are fused into a single chain, denoted EgtUBC encoded by *spd_1642*. Quantitative LMW thiol profiling reveals that both functional EgtUA and EgtUBC are required for ET accumulation in *S. pneumoniae*. The ET-bound crystal structure of EgtUC, coupled with extensive NMR studies, provides novel insights into ET affinity, binding mechanism, and specificity. Bioinformatics analyses and accompanying biophysical studies reveal that EgtU is widely distributed in Firmicutes including the human pathogens *Enterococcus faecalis*, *Staphylococcus aureus*, and *Listeria monocytogenes*, the latter as a bile acid exclusion and virulence determinant^29^. This discovery expands the diversity of the LMW thiols to include ET in an important pathogen where it may contribute to antioxidant defenses in the infected host.

## Results

### *Spd_1642-1643* encodes an ABC transporter specific for ET

**Fig. 1:**
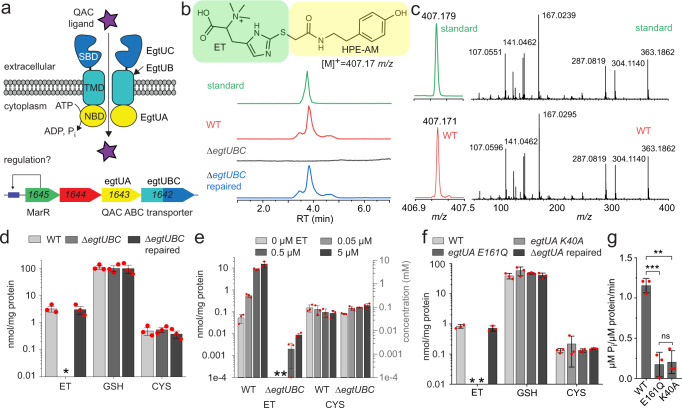
*spd_1643-1642* encodes an ET uptake transporter in *S. pneumoniae* denoted EgtU. **a** Hypothesized functional roles of the genes of the streptococcal conserved operon^30^ harboring a candidate QAC ABC transporter, encoded by *spd_1642-1643*. **b** Structure of HPE-IAM-derivatized (capped) ET and LC traces of capped ET from authentic ET (standard) and cell lysates from the indicated *S. pneumoniae* D39 strains grown on BHI media. WT, wild-type; ∆*egtU*, markerless deletion of most of the *egtU* gene, which was then repaired via insertion of a wild-type *egtU* allele (∆*egtU* repaired). **c** MS1 (*left*) and LC-MS/MS (*right*) spectra of HPE-IAM capped ET found in a wild-type (WT) cell lysate from cells grown in BHI vs. authentic ET (standard). **d** Normalized content of ET, GSH and CYS found in the indicated strains of *S. pneumoniae* D39 grown in BHI in biological triplicate. *, not detected (≤0.0001 nmol thiol/mg protein). **e** Normalized content of ET and CYS in the indicated strains of *S. pneumoniae* D39 grown in CDM supplemented with indicated concentration of ET in biological triplicate. *, not detected (≤0.001 nmol/mg protein). GSH is not detected in these cell lysates (≤0.0001 nmol/mg protein). Molar thiol concentrations estimated from nmol/mg protein in *S. pneumoniae* as described^88^ (see also Supplementary Fig. 5). **f** Same as **d** but with the indicated *egtUA* mutant strains. **g** ATPase activity of WT vs. mutant EgtUA proteins in vitro. **p < 0.01, ***p < 0.001 in a one-sided t-test. Data for **d**, **e**, **f**, and **g** are shown as the mean and standard deviation of three independent replicates, with individual measurements shown as red circles. Source data for **d**, **e**, **f** and **g** are provided as a Source Data file.

We recently identified an uncharacterized operon in *Streptococcus pneumoniae* D39, *spd_1642-1645*, that is highly conserved in *Streptococci* and is regulated in part by a quinone-sensing Rrf2 family transcriptional regulator, SifR^30^. The SifR regulon allows access to a host-derived nutritional catechol-iron source, while avoiding oxidative and electrophile stress-associated catechol oxidation^30^. This operon encodes an uncharacterized MarR family transcriptional regulator^31^ (*spd_1645*), a putative Snoal2 family polyketide cyclase/hydrolase^32^ (*spd_1644*) and an ABC transporter annotated as an osmoprotectant uptake system (Opu or Pro) that transports quaternary amines, e.g., glycine betaine (GB) or *L*-proline (*spd_1642-1643*) (Fig. 1a)^33^. Given the connection of SifR to redox stress and iron assimilation, we hypothesized that SPD_1642 is involved in ET uptake and thus named this *spd_1642-1643* cluster *egtU* (ergothioneine uptake), where *spd_1642* encodes EgtUBC and *spd_1643* encodes EgtUA.

To test this hypothesis, we used a mass spectrometry-based thiol profiling strategy to quantify LMW thiols present in lysates obtained from exponentially growing *S. pneumoniae* cells (Fig. 1b, c; Supplementary Figs. 1–4). With this approach, isotopically labeled LMW thiol standards are spiked in at a known concentration and used to quantify the concentration of LMW thiols in the cell lysate samples. We find that glutathione (GSH)^34^, cysteine (Cys) and ET are major LMW thiols in a wild-type *S. pneumoniae* D39 strain cultured in a brain-heart infusion (BHI) rich growth medium (Fig. 1d). Moreover, a markerless ∆*egtUBC* strain lacks detectable ET, while ET levels are restored in an *egtUBC*-repaired strain (Fig. 1d). In strong contrast, cellular levels of GSH and cysteine are unaffected by the loss of *egtUBC*. We next quantified pneumococcal thiol levels when grown in a chemically defined medium to which variable ET was added (0.05–5 µM). These studies reveal a concentration-dependent increase in cellular ET that is lost in the ∆*egtUBC* strain, with no impact on Cys levels and no detectable GSH (Fig. 1e). Furthermore, two independent *egtUA* mutant strains derived from a ∆*egtUA* parent strain that express mutant EgtUAs with no in vitro ATPase activity (Fig. 1g) fail to import ET into cells (Fig. 1f) in a way that can be rescued by reintroduction of the wild-type *egtUA* allele. This experiment reveals that ATP hydrolysis is required to concentrate ET against a concentration gradient, to ≈1 mM in cells (Fig. 1e; Supplementary Fig. 5). These findings collectively show that EgtU is an ergothioneine-specific uptake ABC transporter in *S. pneumoniae* with *spd_1642* encoding a transmembrane permease domain-solute binding domain (TMD-SBD) fusion protein, EgtUBC, and *spd_1643* encoding the ATPase EgtAU required to power uptake.

### The EgtU SBD binds ET with high affinity

**Fig. 2:**
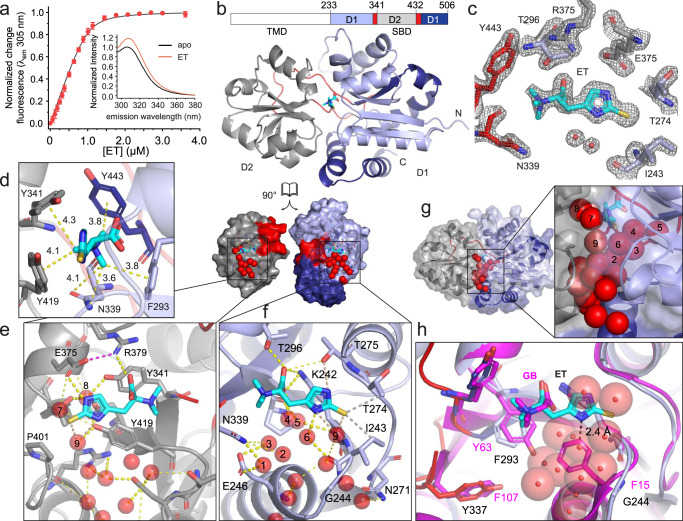
Structure of ET-bound *Sp*EgtUC. **a** Titration of ET into 1.0 µM *Sp*EgtUC monitored by change in intrinsic Tyr fluorescence. Each titration point is shown as the mean and standard deviation of three independent replicates. *Inset*, tyrosine emission spectra of *Sp*EgtUC in the absence (black) and presence (red) of saturating ET. **b** Crystal structure of ET-bound EgtUC_CTT_ shown as ribbon, with D1 shaded light blue (residues 232–331) and dark blue (445-503), D2 shaded gray (341-432) and linkers colored red. ET is shown as cyan sticks. **c** Electron density map of ET and surrounding residues in the ligand binding pocket. **d** Quaternary amine region of the ET binding pocket, with residues in the aromatic pentagon shown as sticks, with polar and cation–π interactions shown as yellow dashed lines, with distances shown in Å. **e** ET binding pocket of D2, with backbone ribbon colored as in **a**. Water molecules within 4 Å of heavy atoms are shown as red spheres. Side chains in contact with water molecules or ET are shown as sticks. Polar interactions less than 4 Å are shown as yellow dashed lines. **f** The D1 ET binding pocket displayed as in **d**, with C-H•••S hydrogen bonds shown as gray dashed lines (Supplementary Fig. 9c). **g** High-occupancy water molecules (a subset are labeled 1-9 in panels **d** and **e**; see Supplementary Table 3) lining the binding pocket and interdomain cleft, shown as red spheres. **h** Overlay of ligand binding pockets from the GB SBP *Af*ProX (magenta, PDB 1SW2) and *Sp*EgtUC (blue/red/cyan), showing that a conserved G244 in *Sp*EgtU SBD provides space for a chain of water molecules (red spheres). The F-to-Y switch between EgtU and *Af*ProX homologs is also labeled, with F293 and Y337 in *Sp*EgtUC and Y63 and F107 *Af*ProX shown as sticks.

EgtUBC is predicted to contain six transmembrane helices in residues 1-230 (EgtUB), followed by the soluble, extracellular substrate-binding domain (EgtUC, Supplementary Fig. 6). Recombinant EgtUC can be expressed alone (residues 233–506), with high yield, purity, and hydrodynamic homogeneity (Supplementary Fig. 7a). This construct is thermally stable, with a melting temperature of 52 °C (Supplementary Fig. 8a, b). Perturbation of intrinsic tyrosine fluorescence (EgtUC has no Trp residues) is a convenient technique for measuring ligand binding in vitro, and has been previously applied to other QAC-binding proteins^35,36^. The intrinsic tyrosine fluorescence of purified EgtUC increases upon addition of ET, accompanied by a slight red shift in the emission spectrum (Fig. 2a). These data confirm that EgtUC binds ET as a 1:1 complex, and reveal a *K*_a_ of ≈2.0 × 10^7 ^M^−1^ (Table 1), comparable to that of *E. coli* HisJ for histidine and a number of other SBP-ligand complexes^28,37^. The perturbation of the tyrosine fluorescence suggests that EgtU may engage ET by trimethylamine cation-π interactions in a manner analogous to SBPs specific for osmoprotectants GB and choline^38,39^.

**Table 1:** Summary of parameters obtained for the binding of ET and other ligands to wild-type and mutant *Sp*EgtUC

SBD	ligand	Method^a^	*K*_a_ (M^−1^)	∆*G* (kcal mol^−1^)	∆*H* (kcal mol^−1^)	–*T*∆S (kcal mol^−1^)	*n*
*Sp*EgtUC	ET	TYR	2.0 ± 0.1 × 10^7^	–10.0 ± 0.1	–	–	
F277W/L374C- bimane *Sp*EgtUC	ET	PiFQ	2.0 ± 0.4 × 10^7^	–9.9 ± 0.1	–	–	
*Sp*EgtUC	ET	ITC	1.7 ± 0.1 × 10^7^	–9.9 ± 0.1	–10.4 ± 0.1	0.5 ± 0.1	0.99 ± 0.01
*Sp*EgtUC_CTT_	ET	ITC	1.9 ± 0.1 × 10^7^	–9.9 ± 0.1	–10.7 ± 0.4	0.8 ± 0.4	0.95 ± 0.04
*Sp*EgtUC-GFP	ET	GFP	1.2 ± 0.2 × 10^7^	–9.7 ± 0.1	–	–	
*Sp*EgtUC-GFP	HER	GFP	5.2 ± 0.5 × 10^3^	–5.1 ± 0.1	–	–	
*Sp*EgtUC	HER	NMR	6 ± 1 × 10^2^	–3.8 ± 0.1	–	–	
*Sp*EgtUC	GB	NMR	<30	<–2.0	–	–	
G244F *Sp*EgtUC	ET	ITC	2.0 ± 0.3 × 10^5^	–7.2 ± 0.1	–8.2 ± 0.3	1.0 ± 0.2	0.88 ± 0.01
Y419F *Sp*EgtUC	ET	ITC	1.6 ± 0.1 × 10^6^	–8.5 ± 0.1	–7.3 ± 0.5	–1.2 ± 0.4	0.91 ± 0.02
F293Y *Sp*EgtUC	ET	ITC	2.0 ± 0.1 × 10^6^	–8.6 ± 0.3	–9.9 ± 0.2	1.3 ± 0.2	1.06 ± 0.03
E375Q *Sp*EgtUC	ET	ITC	4.0 ± 0.2 × 10^4^	−6.3 ± 0.1	–^b^	–^b^	0.75 ± 0.03
*Ef*EgtUC	ET	TYR	1.6 ± 0.3 × 10^7^	–9.8 ± 0.1	–	–	
*Sa*EgtUC	ET	TYR	4.6 ± 1.6 × 10^6^	–9.1 ± 0.3	–	–	
*Lm*EgtUC	ET	TYR	1.8 ± 0.8 × 10^7^	–9.9 ± 0.4	–	–	

^a^Methods: TYR: measured using intrinsic Tyr fluorescence enhancement; PiFQ, measured by monitoring the quenching of bimane fluorescence by a nearby Trp residue; ITC, isothermal titration calorimetry; GFP, measured using the *Sp*EgtUC-GFP fusion protein; NMR, measured by monitoring chemical shift perturbations by NMR spectroscopy. Conditions for NMR: 10 mM sodium phosphate pH 7.0, 150 mM NaCl, 35.0 °C. Conditions for all other methods: 50 mM HEPES, pH 7.5, 150 mM NaCl, 2 mM EDTA, 25.0 °C, with the parameter values from two or three independent experiments shown as the mean and standard deviation.

^b^Binding affinity too low (*c* value = 1.2) for accurate ∆*H* and ∆*S* fitting under these conditions.

### Crystal structure of the EgtUC-ET complex

To identify the molecular determinants of ET binding by the EgtUC domain, we determined the atomic structure using X-ray crystallography (Fig. 2b). Two structures of EgtUC-ET complexes were independently obtained, at 1.82 and 2.44 Å resolution (Supplementary Table 1). The 2.44 Å structure of the holo EgtUC contains the wild-type EgtUC residues 233-506, while the 1.82 Å structure, termed EgtUC_CTT_, has the C-terminal five residues, GLLKK, replaced by the pair of amino acids VC. Isothermal titration calorimetry shows that EgtUC_CTT_ has ET binding affinity and thermodynamics that are identical to the wild-type protein (Supplementary Fig. 9a, Table 1). The structures are virtually identical with a pairwise heavy-atom RMSD of 0.206 Å over the common regions (residues 233–501; Supplementary Fig. 9b), and we therefore use the higher resolution EgtUC_CTT_-ET structure to describe its features. The structure of EgtUC includes two globular subdomains connected by a hinge consisting of two strands ≈10 residues long, with the ligand bound in the cleft between subdomains. The domain D1 consists of residues 233-331 and C-terminal residues 445–506, while D2 encompasses residues 341–432 (Fig. 2b). Each domain is characterized by a five-stranded β-sheet surrounded by five or six α-helices. The additional electron density found in the cleft between two domains corresponds precisely to that of *L*-ET (Fig. 2c), with the quaternary amine oriented toward the hinge and the bulky sulfur atom close to opening of the binding pocket. Given the short C-S bond in bound ET (1.596 Å), we conclude that it is the thione tautomer that is bound by EgtUC (Supplementary Fig. 1).

The long, two-stranded hinge between domains identifies EgtUC as a type II SBP^40,41^, and more specifically as a member of cluster F^28^. EgtUC belongs to subcluster F-III, which employs a conserved set of aromatic residues arranged in a cage-like structure to coordinate the quaternary amine of the histidine betaine moiety. As in some other subcluster F-III quaternary ammonium compound (QAC) binding proteins, notably *Archaeoglobus fulgidus* ProX (specific for GB)^35^, *B. subtilis* OpuBC (choline)^42^ and *B. subtilis* OpuCC (broad QAC substrate specificity)^43^, the aromatic residues Y341, Y419, Y443 and F293 form four sides of a pentagon and contribute cation-π interactions, while the base of the pentagon is formed by N339 (Fig. 2d). Three of these five residues, N339, Y341, and Y443, are within the two interdomain linkers. The carboxyl group of ET makes electrostatic interactions with K242, T296 and R379 (Fig. 2e, f).

The thioimidazole moiety of ET protrudes from the pentagonal cage toward the opening of the ET binding cleft, with the imidazole ring aligned roughly parallel to the domain interface. Here, E375 engages N^ε2^ in a hydrogen bonding interaction (Fig. 2e), while the hydroxyl group of T275 is close to N^δ1^ (Fig. 2f). The side chain of I243, the H**α** and methyl group of T274, and the aliphatic region of the K242 side chain make van der Waals contact with the thione S of ET and all appear to meet the definition of a C-H•••S hydrogen bond^44^ (Supplementary Table 2, Supplementary Fig. 9c). Except for T274, these residues are strongly conserved among EgtU sequences. The orientation of ET in the binding pocket is strikingly similar to that of histidine in the HisJ binding pocket, despite a lack of similarity in interacting residues (Supplementary Fig. 10a)^37^. The cation-π interactions stabilize the trimethyl ammonium moiety in a similar part of the binding pocket as those of GB or choline in *Af*ProX^35,45^, or OpuBC^42^ and OpuCC^43^ from *B. subtilis*, although the latter two rely on hydrogen bonding interactions with the protein backbone rather than a salt bridge to a conserved lysine sidechain to orient the carboxylate (Supplementary Fig. 10b). In contrast, the carboxylate of ET is oriented in an entirely different direction from that of GB in SBDs that use Trp sidechains for cation-π interactions with the trimethyl amine moiety of GB, such as is OpuAC from *L. lactis* or *B. subtilis* or ProX from *E. coli* (Supplementary Fig. 10c)^39,45,46^.

A string of highly ordered, high occupancy water molecules appears to surround the thioimidazole ring, making close contacts with the thione S and imidazole N^ε2^, while also bridging conserved tyrosines Y419 and Y341 from each of the two domains (Fig. 2e–g; Supplementary Table 3). This network is connected to surface waters positioned in the cleft between the two domains. G244 is near these water molecules (Fig. 2f) and is invariant in EgtU SBDs (see below). In the GB-binding SBP *Af*ProX G244 is replaced with phenylalanine, which would severely disrupt the buried water molecules (Fig. 2 h). While all *Af*ProX-family GB-specific SBPs use four Tyr to create the pentagonal cage, all EgtUs have F293 in place of *Af*ProX Y63, which is accompanied by a switch of Y337 for F107 in *Af*ProX (Fig. 2h).

### Mutations of key residues impact ET binding affinity and thermodynamics

**Fig. 3:**
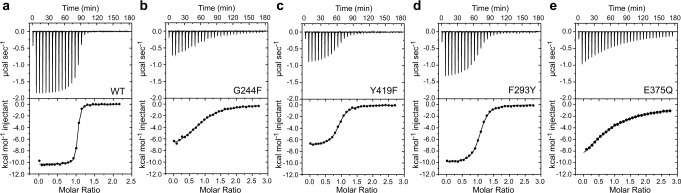
Thermodynamics of ET binding to wild-type and mutant *Sp*EgtUCs. **a–e** Thermograms and representative ITC-derived ET binding curves obtained for WT and indicated mutant EgtUCs. The continuous lines show the best fit to a single-site binding model. Thermodynamic parameters are compiled in Table 1. Each titration shown is representative of at least two independent replicates. Source data are provided as a Source Data file.

In some SBPs, water molecules that line the binding pocket have been proposed to contribute to ligand promiscuity^47^, while in others^48^, they are thought to contribute to an enthalpic driving force for binding via H-bonding while also enhancing ligand selectivity. We therefore used isothermal titration calorimetry (ITC) to measure the thermodynamics of ET binding to EgtUC (Fig. 3a) and to assess the impact of mutations in perturbation of the global energetics of binding. ET binding is strongly enthalpically driven, with a ∆*H* comparable to ∆*G*, and a small unfavorable *T*∆*S* value (Fig. 3a, Table 1). These thermodynamic parameters are rather similar to those previously found for the histidine-HisJ complex^37^, which suggests that trimethylamine cation-π interactions with Tyr/Phe are not necessarily a major net contributor to the ∆*H* term in SBP-QAC ligand complexes^49^. The G244F substitution mutant binds ET ≈ 100-fold more weakly than wild-type EgtUC, with a far less favorable ∆*H*, potentially consistent with a perturbation of the water network (Fig. 3b). However, the thermal stability of G244F EgtUC is significantly reduced, consistent with a global impact on structure; nonetheless, the stability of this mutant is only marginally rescued by ET (Supplementary Fig. 8a–c).

Two other substitutions that target conserved residues Y419 (Y419F) and F293 (F293Y) give rise to somewhat smaller, but readily detectable perturbations of the binding energetics and the affinity (Fig. 3c, d). Consistent with this, differential scanning fluorimetry reveals that ET stabilizes these two mutants against thermal denaturation, but to an extent less than that of WT EgtUC (Supplementary Fig. 8b, d, f). E375, in contrast, appears intimately involved in a number of key interactions beyond an H-bond with N^ε2^ of ET, including a salt bridge with R379, the side chain of which becomes strongly ordered upon ET-binding (see below), and an H-bond to the W7 water molecule (Fig. 2e). We find that a sterically conservative E375Q substitution reduces the binding affinity for ET ≈ 500-fold (Fig. 3e; Table 1); the thermal stability of E375Q EgtUC is identical to wild-type EgtUC and is virtually unaffected by ET-binding (Supplementary Fig. 8b, e). These findings parallel the impact of the analogous Glu-to-Ala substitution in *Af*ProX^35^ on GB binding affinity, but the E375Q substitution is comparatively more destabilizing to the ET–EgtUC complex.

### ET induces a significant conformational change in EgtUC

**Fig. 4:**
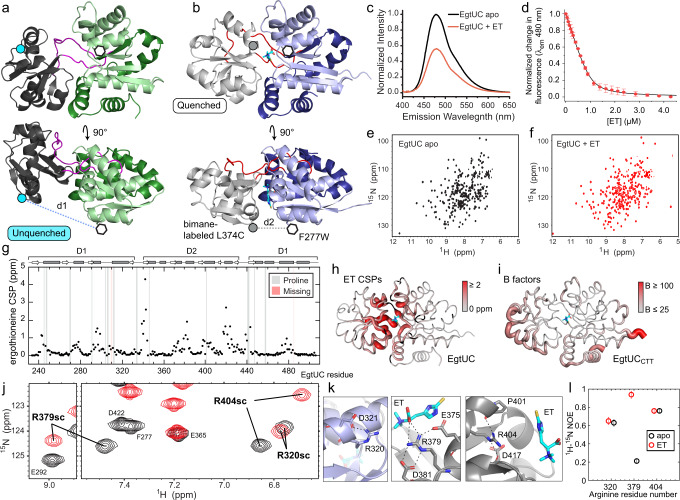
Conformational and dynamic changes in *Sp*EgtUC upon ET binding. **a** AlphaFold2 model of “open” apo *Sp*EgtUC (gray D2, green D1, magenta linkers). F277W and the L374C site of bimane labeling are shown as an open hexagon and a cyan circle, respectively. **b** The “closed” crystal structure of ET-bound SBD (gray D2, blue D1, red linkers), with F277W and L374C shown as in **a**. **c** Bimane fluorescence emission spectra of bimane-labeled L374C/F277W *Sp*EgtUC with (red) and without (black) ET. **d** Normalized change in fluorescence emission at 480 nm upon titration of bimane-labeled L374C/F277W *Sp*EgtUC with ET in a position-induced fluorescence quenching (PiFQ) experiment^51^. Each titration point is shown as the mean and standard deviation of two independent replicates. See Table 1 for fitted parameters. **e** ^1^H,^15^N TROSY spectrum of apo EgtUC (residue-specific assignments in Supplementary Fig. 12). **f** ^1^H,^15^N TROSY spectrum of EgtUC bound to equimolar ET (Supplementary Fig. 13). **g** Backbone chemical shift perturbations (CSPs) upon binding ET for each residue in *Sp*EgtUC. Assignments are missing for residues H310 and V485 in the ET-bound state (shaded pink). Prolines are shaded gray. **h** CSPs of ET binding painted onto the crystal structure of ET-bound *Sp*EgtUC, with large chemical shift changes shown as thick, red tubes. **i** B-factors plotted on the crystal structure of *Sp*EgtUC_CTT_, with high values shown as thick, red tubes, revealing low B-factors in the interdomain linkers, and comparable to the B-factors of high occupancy solvent molecules. **j** Overlay of ^1^H,^15^N TROSY spectra of apo and ET-bound EgtUC, zoomed to the region where the arginine side chain peaks are folded into this spectral window. **k** H-bond networks of arginine side chains (R320, R379, R404) found in the crystal structure. **l** ^1^H,^15^N heteronuclear NOEs for these three arginine side chains. Heteronuclear NOE data were recorded as one replicate, with error bars indicating the uncertainty derived from spectral noise. Source data for those data shown in panel d are provided as a Source Data file.

In the absence of a structure of ligand-free EgtUC, we used AlphaFold2^50^ to model the apo state, and found a much more open conformation (Fig. 4a) relative to the “closed”, ligand-bound structure. This model closely resembles the apo-state structure of the homolog *Listeria monocytogenes* BilEB (PDB 4Z7E)^38^, which is 60% identical to *Sp*EgtUC. The individual subdomains D1 and D2 are nearly identical in the apo model and the ET-bound structure. Differences between the phi and psi backbone dihedral angles are limited and largely localized to a few loops and the linkers that connect the domains (Supplementary Fig. 11a). However, these limited changes in the linkers are sufficient such that structural alignment of D1 results in a displacement and a 54° rotation of D2 (Fig. 4a, b).

In order to validate the model and to assess whether ET drives an open-to-closed transition in solution, we prepared a F277W/L374C double mutant of EgtUC, introducing a nonnative Trp in D1, while attaching a bimane group to C374 in D2. Because the EgtU lacks native Trp residues, the bimane fluorescence should be quenched by Trp only when these two residues are in close proximity, termed position-induced fluorescence quenching (PiFQ) (Fig. 4a, b)^51^. Titration with ET results in a significant quenching of the bimane fluorescence (Fig. 4c), and the binding affinity of this construct is identical to that of wild-type EgtUC (Fig. 4d; Table 1), supporting the model of ET-mediated domain closure of the SBD.

### NMR studies of ligand-induced conformational change in EgtUC

In order to understand the differences between the apo- and ligand-bound states of EgtUC in more detail, we turned to NMR spectroscopy. The 2D ^1^H,^15^N TROSY spectrum of apo EgtUC shows broad chemical shift dispersion, with uniform crosspeak intensities, consistent with a globular domain with an α/β fold (Fig. 4e). Addition of equimolar ET causes significant changes in the spectrum (Fig. 4f). Backbone chemical shift assignments of the apo- and ET-bound states of ^15^N, ^13^C, ^2^H-*Sp*EgtUC (Supplementary Figs. 12 and 13) give rise to chemical shift-based secondary structure predictions of the ET-bound state that are identical to the crystal structure, and are strikingly similar in the apo state, consistent with our structural model that shows minimal changes in the individual subdomains. Closer inspection reveals that the apo state has an extended β−strand at the end of the first linker and in a neighboring strand in D2 (Supplementary Fig. 11b). This small structural change in the linker is consistent with the difference between the ET-bound structure and apo model, sufficient to describe the opening and closing of the entire SBD. Chemical shift perturbations (CSPs) caused by equimolar ET binding are dramatic, but nearly exclusively localized to the domain interface (Fig. 4g, h).

We next used NMR spectroscopy to investigate the extent to which ligand binding affects protein flexibility, and in particular whether apo EgtUC samples both open and closed states, in binding ligand via a conformational selection mechanism. As expected, the bound form features low crystallographic B-factors (Fig. 4i) and high ^15^N[^1^H] heteronuclear nuclear Overhauser enhancements (hNOE) throughout, including the linkers, revealing that the linkers are strikingly rigid when bound to ET. Moreover, high hNOEs strongly suggest that the linker is also rigid in the apo state (Supplementary Fig. 11c). Mobility in both states is largely restricted to the termini and to a long loop within D1, consisting of residues 301-312 (Supplementary Fig. 11c–e). These data reveal that the binding of ET to EgtUC has strikingly little impact on sub-ns backbone dynamics throughout the molecule.

^15^N *R*_1_ and *R*_2_ longitudinal and transverse relaxation rates (Supplementary Fig. 11f) are largely similar in both the apo and ET-bound states, revealing relatively slow, anisotropic tumbling. The most notable difference between the two states is in the D2 helix spanning residues 375–390, which has lower *R*_1_ and higher *R*_2_ values in the ET-bound state. *R*_1_ and *R*_2_ rates are sensitive to N-H bond vector orientation as well as sub-ns flexibility, and HYDRONMR^52^ can be used to distinguish whether the difference is due to a change in mobility or simply a change in conformation, by computing theoretical relaxation rates for a rigid body of known structure tumbling in solution with no internal mobility. HYDRONMR was therefore used to predict backbone relaxation parameters for the ET-bound crystal structure as well as for several models of the apo state. At one extreme, we examined a model identical to the ET-bound crystal structure, while at the other extreme, domains D1 and D2 were allowed to tumble independently of one another, connected by an infinitely flexible linker. The AlphaFold2 model is intermediate, rigid but structurally distinct from the ET-bound state.

As expected, HYDRONMR predictions for the *R*_2_/*R*_1_ ratio derived from the ET-bound crystal structure correlate well to the experimental parameters for ET-bound EgtUC in solution (Supplementary Fig. 11g), better than to the experimental parameters for apo EgtUC (Supplementary Fig. 11h). This result is consistent with a rigid bound structure with mobility largely limited to loops. The apo experimental data correlate significantly better to the fully rigid AlphaFold2 model (Supplementary Fig. 11i) than to the ET-bound crystal structure or to the model with fully uncoupled tumbling of the D1 and D2 domains (Supplementary Fig. 11j). A residue-by-residue analysis reveals the main site of flexibility in the bound state is the long D1 loop (residues 301-312) already identified by low hNOEs, with line broadening in the 2D ^1^H,^15^N TROSY spectra as well as reduced *R*_2_/*R*_1_ values (Supplementary Fig. 11k). The apo state predictions match strikingly well to the experimental values (Supplementary Fig. 11l); the differences in *R*_1_ observed in the helix in the middle of D2 (Supplementary Fig. 11f, *upper panel*) appears to derive from a reorientation of the bond vectors relative to the long axis of the molecule. These data strongly suggest that ET binding triggers an induced fit, rigid-body transition from a conformationally narrow open state to another conformationally narrow closed state, in striking contrast to expectations of a conformational selection model.

Although the backbone relaxation parameters in EgtUC are strikingly insensitive to ET binding, side chains in the binding pocket are strongly affected. Arginine guanidino protons are rarely observable in ^15^N,^1^H-TROSY spectra acquired at pH 7.0 due to their high rate of solvent exchange, particularly when not involved in hydrogen bonds. Three slowly exchanging guanidino protons are observable (Fig. 4j), and all form hydrogen bonds in the crystal structure (Fig. 4k). R379, in particular, is sandwiched between the conserved E375 and D381 side chains, and forms a H-bond to the carboxylate oxygen of ET. The hNOEs of the side chains of R320 and R404 are unaffected by ET binding, but the hNOE of R379 is low in the absence of ligand and is dramatically increased upon binding to ET, indicating that motional disorder on the sub-ns timescale is quenched in the presence of ET (Fig. 4l).

### EgtUC binding to ET is highly specific

**Fig. 5:**
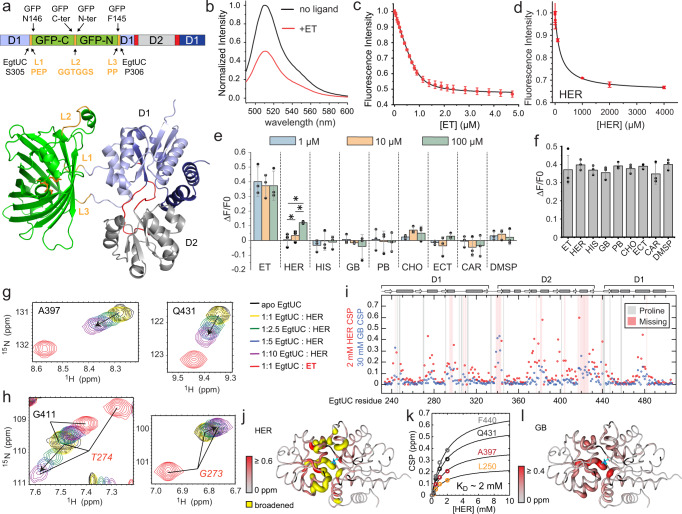
The *Sp*EgtUC binds ET with high selectivity. **a** *Upper*, schematic representation of the EgtUC-GFP fusion protein construct; *lower*, ribbon representation of an AlphaFold2 model of the EgtUC-GFP fusion, with domains indicated. **b** GFP fluorescence emission spectra, *λ*_ex_ = 470 nm with or without 10 µM ET. **c** ET binding to the EgtUC-GFP fusion protein, monitored by quenching of the GFP fluorescence. Each data point is shown as the mean and standard deviation of three independent replicates. Continuous curve, fit to a 1:1 binding model; see Table 1 for binding parameters. **d** same as **c** except *L*-hercynine was added to EgtUC-GFP fusion protein (Table 1). **e** Quenching of GFP fluorescence of the EgtUC-GFP fusion protein following addition of 1, 10, or 100 µM of the indicated ligand (see Supplementary Fig. 14a for chemical structures). HIS, *L*-histidine; PB, proline-betaine; CHO, choline; ECT, ectoine; CAR, carnitine; DMSP, dimethylpropiothetin. Each bar represents triplicate measurements with each data point represented by a filled circle. *p < 0.05 in a one-sided t-test. **f** Same as **e**, except that 1 µM ET (left bar) was compared to a mixture of 1 µM ET and 100 µM of the indicated ligand (other bars). Each bar represents triplicate measurements (filled circles). **g** and **h** Movement of the indicated backbone NH crosspeak from the apo-state (black) as *L*-hercynine (HER) is added (yellow to purple), compared to the cross peak position of ET-bound EgtUC (red). **i** Backbone chemical shift perturbation (CSP) maps resulting from the addition of 2 mM HER or 30 mM glycine-betaine (GB). **j** Backbone CSP maps upon HER binding painted onto the *Sp*EgtUC structure. **k** Global fits of the movement of selected NH cross-peaks as a function of [HER] to a 1:1 binding model are shown (Table 1). **l** Backbone CSPs upon HER binding painted onto the *Sp*EgtUC structure. Source data for **c**–**f**, **i**, and **k** are provided as a Source Data file.

We next wished to critically evaluate the specificity of ligand binding by EgtUC since this is a key feature of the function of EgtU as an ET transporter in cells. We first used differential scanning fluorimetry to show that *L*-hercynine induces only a small, concentration-dependent shift in *T*_*m*_, 0.5 °C at 1 mM, which is far less than that of ET, which increases the EgtUC *T*_*m*_ by nearly 7 °C (Supplementary Fig. 8a, b). Another high-throughput method for exploring the ligand specificity of EgtUC is also a first step to the development EgtUC as a genetically-encoded biosensor, involving the insertion of a circularly-permuted green fluorescent protein (GFP) sequence into EgtUC (Fig. 5a). Analyte binding to the sensing domain induces a conformational change in the GFP at the insertion site, which has been engineered to be near the chromophore, inducing a change in GFP fluorescence^53,54^.

We find that our EgtUC-GFP fusion protein exhibits fluorescence that is strongly quenched upon ET binding (Fig. 5b), and that it binds ET with an affinity similar to WT EgtUC (Fig. 5c; Table 1). The low volume and high sensitivity of this experiment permit quantitative measurement of very low-affinity interactions with minimal consumption of protein and ligand, and we find that *L*-hercynine binds ≈10,000-fold less tightly than ET (Fig. 5d), consistent with the thermal unfolding results. We then used this assay to screen the ability of other potential ligands (Supplementary Fig. 14) to quench the fluorescence of the EgtUC-GFP fusion protein and find that none do so, at concentrations 1000-fold higher than the *K*_d_ for ET (Fig. 5e), nor do they negatively impact the ability of ET to quench the fluorescence of EgtUC-GFP at 100-fold molar excess ligand relative to ET (Fig. 5f). ITC reveals no detectable change in global heat observed for selected other ligands, even for *L*-hercynine which binds weakly despite lacking only the thione sulfur atom of ET (Supplementary Fig. 14d). These experiments establish that our EgtUC-based sensor is highly specific for ET, suggesting that such a fusion protein could be used to monitor ET concentrations inside cells after further optimization^54^.

NMR was next used to probe the binding of low-affinity ligands to EgtUC in more detail. A titration of *L*-hercynine into ^15^N-labeled EgtUC shows that the ligand-bound and free conformations are in fast-to-intermediate chemical exchange on the ^1^H NMR timescale, with most peaks generally moving towards the corresponding resonance frequency of the ET-bound residue (Fig. 5g), while many vanish entirely. Only a few resonances, e.g., G273 and T274, shift in a direction that is opposite to ET (Fig. 5h); these residues are in close proximity to the thione sulfur atom (Fig. 2f). A large molar excess of ligand shows clear evidence of specific binding, with CSPs localized to the same interfacial loops that respond to ET (Fig. 5i, j; Supplementary Fig. 15a), but 2 mM hercynine was insufficient to saturate EgtUC. Fitting the chemical shift perturbations for several residues as a function of ligand concentration gives an affinity estimate of 600 M^−1^ (Table 1, Fig. 5k). Titration of GB reveals only fast chemical exchange behavior, consistent with even weaker binding, as 30 mM GB fails to reach saturation (Supplementary Fig. 15b). Largely the same binding pocket residues are affected in the ET and GB complexes (Fig. 5i, l), but with an affinity estimated to be less than 30 M^−1^ (Table 1).

### EgtU homologs are widely distributed across the genomes of Firmicutes

**Fig. 6:**
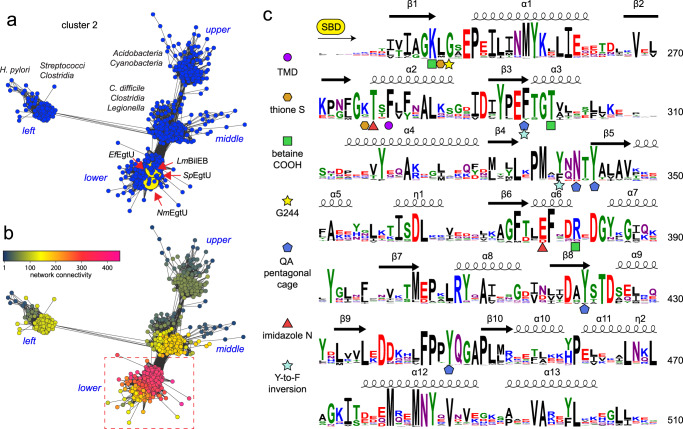
EgtU homologs cluster in a grouping of highly similar sequences within a subcluster of SSN cluster 2. **a** Several EgtU sequence metanodes are highlighted with a large yellow circle, including *L. monocytogenes* BilEB and *E. faecalis* EgtUC biochemically characterized here. The lower (*Sp*EgtU-like), middle, left and upper subclusters of SSN cluster 2 are labeled, with full sequence conservation maps shown in Supplementary Fig. 19. The middle subcluster sequences are derived from anaerobes or obligate anaerobes, including those recently studied in *C. difficile*^57^. **b** Representation of SSN cluster 2 colored according to neighborhood connectivity (NC; see scale), with those sequences within a single metanode that are most closely related characterized by a large NC index (and shaded *magenta*). **c** Sequence logo representation of conservation in the SBD of EgtUs of those closely related sequences encircled by the *red* box in **b**. Secondary structure of the SBD is indicated, based on the structure of *Sp*EgtUC. Residues discussed are highlighted with a specific symbol of interaction, as indicated. See text for additional details. TMD, predicted sites of interaction with the transmembrane domain. Source data for these images shown in panels **a**, **b** are provided as a Source Data file.

We next asked if EgtUCs cluster in a global sequence analysis, while also elucidating conserved features of an EgtUC and how this differs from other osmoprotectant transporters. To do this, we used *spd*_1642 as query to construct a sequence similarity network (SSN) using genomic enzymology tools^55^ to visualize the relationships among EgtU homologs in the context of the entire superfamily of osmoprotectant uptake (*opu*) SBPs/SBDs (Supplementary Fig. 16). We find that EgtU is representative of a distinct subcluster of closely related sequences within SSN cluster 2 (Fig. 6a) that are characterized by the largest neighborhood connectivity of the entire SSN map (Fig. 6b; Supplementary Fig. 17). Remarkably, *Sp*EgtU homologs are found nearly exclusively in Firmicutes and include gastrointestinal tract-resident bacteria, notably *Lactococcus lactis*, and a wide range of human opportunistic pathogens beyond *S. pneumoniae*, including pathogenic *Bacillus* spp., *B. cereus* and *B. infantalis* (previously OpuF^56^), *Enterococcus faecalis*, *Neisseria mucosa*, *Staphylococcus aureus* and *Listeria monocytogenes* (Supplementary Fig. 18). A sequence logo representation of the multiple sequence alignment (Fig. 6c) of the SBD subcluster reveals that all functional features described above, including the aromatic cage and residues that interact with the imidazole and thione sulfur moieties in the *Sp*EgtUC-ET complex, are highly conserved. Plotting the sequence conservation from this alignment onto an AlphaFold2 model of an *Sp*EgtUBC dimer with a single SBD identifies several conserved residues at the interface that likely facilitate SBD docking onto the TMD (Supplementary Fig. 19a). On the other hand, EgtU homologs found in other SSN cluster 2 subclusters do not appear to conserve key ET-specificity determinants defined here (Supplementary Fig. 19b–e)^57^, specifically the Y-to-F switch and G244, each of which contribute significantly to ET affinity in *Sp*EgtUC (Fig. 2h and 3). For example, *Clostridioides difficile* OpuF^56^ in the middle subcluster and others may be specific for another QAC, or exhibit relaxed QAC specificity. Indeed, very recent work in *Helicobacter pylori* reveals that some SSN cluster 2 transporters contribute to cellular ET uptake, but are characterized by a significantly lower affinity for ET relative to *Sp*EgtUC^58^.

As a direct test of our functional grouping of proposed ET transporters, we purified and characterized candidate EgtUCs from *E. faecalis, S. aureus* and *L. monocytogenes*. We find that *Ef*EgtUC binds ET with an affinity comparable to that of *Sp*EgtUC (Supplementary Fig. 20a; Table 1), while NMR spectra of apo- and ET-bound *Ef*EgtUC show similar features that are broadly consistent with comparable conformational changes to those described for *Sp*EgtUC (Supplementary Fig. 20d, e). Since ET is obtained in the diet in animals, these findings with *E. faecalis* EgtU might suggest a competition for ET among resident microbiota and opportunistic pathogens in the GI tract for some as yet unknown physiological advantage. Similar experiments were carried out with *Sa*EgtUC and we find a similar binding affinity, and no detectable binding by ITC to *L*-hercynine (Table 1; Supplementary Fig. 20b). The EgtU homolog from *L. monocytogenes*, denoted BilEB, has long been known to be associated with bile acid resistance^29^ and early work ruled out a role for BilEB in the uptake of choline, carnitine or GB^38^. We show here that *Lm*BilEB binds ET with an affinity identical to that of authentic *Sp*EgtUC (Table 1; Supplementary Fig. 20c), which argues strongly that BilEB is an ET uptake transporter.

## Discussion

In this work, we show that *Sp*EgtU possesses characteristics of a bacterial ABC transporter that is specific for the low molecular weight thiol/thione *L*-ergothioneine (ET). We show that deletion of *S. pneumoniae* *spd_1642* or introduction of an ATPase-inactive allele of *spd_1643* creates a strain that is unable to accumulate ET either when grown in a vertebrate tissue-derived growth medium that contains significant endogenous ET, or on a chemically defined medium to which ET has been added. Our studies reveal that EgtUC exhibits high selectivity for ET over even closely related QACs, e.g., L-hercynine. This functional assignment of EgtU as an ET transporter is supported by biochemical experiments on EgtU homologs from three other Firmicutes.

Detailed NMR experiments show that the linkers between subdomains D1 and D2 of *Sp*EgtUC change conformation upon binding to ET but are strikingly rigid in both the apo and bound states on the sub-ns timescale, consistent with an induced-fit model of ligand binding that characterizes many other substrate binding proteins^59^. Such large rotations of one subdomain relative to the other have been observed in a number of other SBPs, including DppA^28,60^. Meanwhile, non-cognate QACs simply fail to stably close the ligand binding cleft between the two subdomains, a remarkable finding given that hercynine differs from ergothioneine only by the loss of the thione S. An extensive network of ordered water molecules may well play an important role in ET complex formation, while several C-H•••S H-bonds^44^ and a hydrogen bond between the conserved E375 sidechain and the protonated N^ε2^ of ET clearly contribute to the ability of EgtUC to distinguish ET from hercynine.

A comparative sequence analysis suggests that EgtU is broadly distributed among Firmicutes known to colonize the vertebrate gastrointestinal (GI) tract, including commensals and pathogens, as well as pathogens known to infect other tissues but also capable of replicating in immune cells. Indeed, a recent report shows that the gut commensal bacterium, *Lactobacillus reuteri*, takes up extracellular ET, although the mechanism of uptake was not defined in that study^61^. It is well established that commensals resist colonization by pathogens in the gut by depleting essential nutrients and remodeling resource allocation in this niche^62^. It is also known that ET can be catabolized by various bacteria, impacting ET bioavailability in their respective niches^58,63^. The work reported here raises the possibility of a competition between commensals and pathogens for a nutrient that may well be protective against oxidative and antibiotic stressors, for which there is now evidence in *Helicobacter pylori*^58^.

Our findings clearly establish a mechanism by which a bacterium need not synthesize ET in order to access its potential antioxidant properties^64^. In this case, ET is likely obtained in the diet of the vertebrate host where GI-resident bacteria that express EgtU would have initial access to this metabolite. As described above, the human ET transporter (ETT) is expressed in a wide range of tissues and cells, including neutrophils and macrophages;^21^ this suggests that ET may be bioavailable to both extracellular and intracellular pathogens, e.g., those phagocytosed by neutrophils. This is not yet known with certainty since concentrations of ET itself have not been comprehensively mapped in a wide variety of tissues or cells in an infected host using the analytical approaches we describe here. However, bacteria found in either the intracellular or extracellular lifestyle may well be capable of capturing ET, given that ETT is reported to transport ET with a *K*_m_ of 20 µM^23^, which is ≈500-fold weaker than the *K*_d_ for bacterial EgtUC described here^58^. Of course, the *K*_m_ for transport by EgtU may not correlate with the *K*_d_ for substrate binding by EgtUC, and this is an important focus of future work.

What role ET plays in bacterial cell physiology can be hypothesized from literature published prior to the knowledge that EgtU homologs encode an ET transporter. For example, deletion of *egtUBC* gives rise to a fitness defect in a lung infection model in *S. pneumoniae* D39 strain and is thus a virulence factor^33^. We provide evidence to suggest that ET is the long-sought metabolite that is transported by *Listeria monocytogenes* BilEB, required to promote an adaptive response to bile acid stress during gastrointestinal transit^29^. How ET protects *L. monocytogenes* from bile acid stress is unknown but oxidative stress resistance is a strong possibility. In methicillin-resistant *S. aureus*, *egtUBC* expression is induced ≥15-fold after long exposures to human neutrophil-derived azurophilic granule proteins, but with no significant response to peroxide and hypochlorous acid stress at the same time points;^65^ this suggests an as yet unknown ET-dependent phagocytosis resistance mechanism to killing by these effectors. In *E. faecalis*, *egtUA* and *egtUBC* are among the most highly upregulated genes in a mouse model of colitis when colonized with a simplified human microbiome, but this increase in expression is lost when *E. faecalis* is monocolonized^66^. This finding suggests that competition for this thiol may be physiologically important^66^. Finally, although recent studies show that EgtU (denoted OpuF) from *Bacillus infantis* and *Bacillus panaciterra*, is capable of rescuing an osmoprotectant uptake-deficient *B. subtilis* strain grown in hyperosmotic conditions, the concentrations required to do this are in the high μM to mM range on a chemically defined growth medium^56^. This finding is consistent with the very weak, but measurable (*K*_d_ ≈ mM) binding of EgtUC to GB and *L*-hercynine observed here. On the other hand, we have not yet elucidated the potential impact of oxidation or methylation of the ET thione S to create sulfonylated ET, or *S*-methylated ET, respectively^67^, or insertion of an oxygen atom to the C5-position to create 5-oxo-ET^9^, or Se substitution of the S atom in ET in selenoneine^68,69^, on EgtUC ligand binding affinity. Indeed, the ligand specificity of EgtU homologs in other SSN cluster 2 SBDs remains to be experimentally validated^57^.

Beyond the function of ET itself, the mechanism by which *egtUABC* is upregulated may also provide insights into the pathogen response to host effectors, especially in oxidative stress adaption. In *M. tuberculosis*, the biosynthesis of ET is regulated by the ROS- and RNS-sensing 4Fe-4S cluster transcriptional regulator WhiB3, with the bacterial concentration of ET increasing ≈7 fold in a ∆*whiB3* strain^64^. In addition, the ET level is significantly increased in ∆*whiB3* when fatty acids serve as the nutritional carbon source^64^. Although ET is present at lower concentrations than the major LMW thiol in *M. tuberculosis*, mycothiol, ET becomes significantly elevated in a mycothiol-biosynthesis deficient strain. Our LMW thiol profiling also confirms that ET is present at a significantly lower cellular level relative to glutathione in *S. pneumoniae*, but comparable to that of cysteine. Distinct from other organisms, *S. pneumoniae* is totally dependent on scavenging glutathione through the ABC transporter GshT from its immediate microenvironment to meet cellular needs^34^. How the pneumococcus and other Firmicutes balance EgtU-mediated uptake of ET vs. other thiols is unknown. How the pneumococcus regulates *egtU* expression is also not yet known, although an uncharacterized dithiol-containing MarR (encoded by *spd*_*1645*) is found in the operon harboring *egtU* (Fig. 1a) whose expression is clearly tied in some way to quinone-derived oxidative stress, mediated in part by catechol-Fe^III^ uptake^30^. The known ability of ET to form coordination complexes with Fe^II^ and Cu^I/II^ may suggest a role in suppressing redox cycling of one or both metals by host-derived H_2_O_2_ and other potent ROS and RNS or in some other role in colonization or virulence^11,70–72^.

## Methods

### Reagents

*L*-glutathione (GSH) was obtained from Sigma Aldrich (G4251), *L*-ergothioneine from Santa Cruz Biotechnology (sc-200814), *L*-cysteine from Fisher Biotech (BP376-100), *L*-hercynine from Toronto Research Chemicals (H288900), *L*-histidine from Sigma Aldrich (H8000), choline chloride from Sigma Aldrich (C1879), glycine-betaine from Sigma Aldrich (B3501), ectoine from Sigma Aldrich (81619), proline-betaine from VWR (TCS0358), *L*-carnitine from Sigma Aldrich (C0283), and dimethypropiothetin hydrochloride from Sigma Aldrich (80828). These chemicals were used without further purification. Other chemicals include IPTG from GoldBio (2481C100), TCEP from Chem-Impex (00194), dithiothreitol from Chem-Impex (00127), Tris-HCl from MP Biomedicals (816100), HEPES from Chem-Impex (00174), EDTA from VWR Chemicals (BDH9232), NaCl from VWR Chemicals (BDH9286), Imidazole from Chem-Impex (00418), Sypro Orange from Sigma (S5692), and monobromobimane (mBBr) from Sigma (B4380). BHI broth was obtained from BD (37500, lot 1159859) while Luria broth was obtained from Fisher Bioreagent (BP9723). Milli-Q water was used to make all solutions.

### *Streptococcus pneumoniae* D39 mutant strain preparation and growth conditions

The mutant strains listed in Supplementary Table 4 were constructed using standard laboratory practices for allelic replacement in *S. pneumoniae* serotype 2 D39W (IU1781)^73^. All mutant strain constructs were sequence verified. Primers are listed in Supplementary Table 5.

### LMW thiol profiling in *Streptococcus pneumoniae* D39

Bacterial cell pellets for LMW thiol profiling were prepared by inoculating selected strains in BHI medium from overnight cultures under microaerophilic conditions with 5% CO_2_ at 37 °C^74^. In a chemically defined medium (CDM)^75^ cells were grown in triplicate in the same condition with or without addition of ET to the indicated concentration (0, 0.05, 0.5, 5 μM). Cells from 5 mL culture were collected at OD_620_ ~0.2-0.3. Cell pellets from 4 mL of this culture were extensively washed in chilled PBS and frozen in –80 °C for LMW thiol profiling. Cell pellets from the remaining 1 mL were washed with chilled PBS and immediately frozen at –80 °C for cellular protein quantification.

Heavy (*D*_4_) HPE-IAM was used to create alkylated selected LMW thiol standards as described^76^. The cellular LMW thiols were alkylated by light (H_4_) HPE-IAM and quantified by LC-MS by spiking a known concentration of LMW thiol derivatized by heavy *D*_4_-HPE-IAM as a standard. *D*_4_-HPE-IAM and HPE-IAM were chemically synthesized as described and structural integrity confirmed by NMR spectroscopy^77^. In brief, each cell pellet was resuspended in 100 µL Milli-Q water with 5 mM HPE-IAM by adding 1 μL 0.5 M HPE-IAM stock prepared in DMSO. The resuspended cell pellet was lysed using a 1 min freeze in liquid N_2_ and 37 °C water bath thaw for 1 min. Five freeze-thaw cycles were performed and cell lysates were further incubated at 37 °C for 1 h, then microcentrifuged at top speed for 20 min. 50 μL supernatants were transferred and filtered by 0.2 μm cutoff micro-centrifuge filter tubes. Then 1 µM heavy *D*_4_-HPE-IAM-derivatized LMW thiol standards (ET, cysteine, GSH) were added into the flow through with total volume brought up to 100 µL with Milli-Q water. Both light and heavy *D*_4_-HPE-IAM labeled standards were prepared by capping 100 μM reduced LMW thiols (ET, cysteine, GSH) with 3 mM *D*_*4*_-HPE-IAM in the lysis buffer at 37 °C for 1 h (see Supplementary Fig. 1). The samples were analyzed by a C18 (YMC-Triart C18) LC system coupled to a Waters SYNAPT G2S high-resolution MS using a mobile phase A (0.25% acetic acid, 10% methanol) and mobile phase B (0.25% acetic acid, 90% methanol) with the following LC elution gradient: 0-3 min, 100% A, 0% B; 3–7 min, linear gradient to 75% A, 25% B; 7–9 min, 75% A, 25% B; 9–12 min, linear gradient to 25% A, 75%B; 12–14 min, linear gradient to 0% A, 100% B; 14–20 min, 0% A, 100% B. The resulting total ion chromatogram (TIC) was searched for positively charged ions (*z* = 1; M^+^ or M + H^+^) (mass tolerance of ±0.02 *m/z;* Supplementary Fig. 1) using Waters MassLynx software and the extracted ion chromatograms of each light (H_4_) and heavy (*D*_4_) HPE-IAM-capped thiol identified in MS1 obtained, peak areas quantified, and identity confirmed by LC-MS/MS by comparison to the corresponding authentic compound standard (Supplementary Figs. 2–4). The ratio of the light and heavy MS1 features was used to calculate the concentration of each thiol using the known concentration heavy standard spiked into the mixture. The remaining 1 mL culture cell pellets were analyzed by Bradford Assay to quantify the total protein concentration of each sample. The LMW thiol concentration is presented as nmol thiol/mg total protein, and where indicated, used to estimate the cellular concentrations (µM) as described in the legend to Supplementary Fig. 5.

### Cloning, protein expression, and purification of EgtUCs from *S. pneumoniae*, *Enterococcus faecalis*, *Staphylococcus aureus*, and *Listeria monocytogenes*

The region of the gene encoding the soluble, extracellular EgtUC domain of *S. pneumoniae* D39 EgtUBC (locus tag *spd_1642*) from residue E233 was PCR-amplified from the genomic DNA. The same was done for the candidate EgtUCs of *E. faecalis* OG1RF EgtU (locus tag *OG1RF_RS02210*) beginning at residue K233, *S. aureus* FPR3757 USA300 (locus tag *sausa300_0707*) beginning at residue G233, and *L. monocytogenes* strain 10403 S (locus tag *lmo1422*) beginning at residue S231. The primers used in the cloning are listed in Supplementary Table 5. Each gene was inserted into the pSUMO expression vector with an N-terminal hexa-histidine tag. All mutants were prepared by PCR-based site-directed mutagenesis. The *Sp*EgtUC-GFP expression construct was prepared using primers (Supplementary Table 5) largely following a published procedure^54^. In brief, the PCR fragment F1 containing *Sp*EgtUC and pSUMO plasmid (6.5 kB) was amplified using *Sp*EgtUC pSUMO expression vector as template and primers *Sp*EgtU_S_P1 and *Sp*EgtU_S_P2. PCR fragments containing the CTD or NTD of “superfolder” GFP (GFP) with linkers were prepared by using primer pairs *Sp*EgtU_S_P3/*Sp*EgtU_S_P4 and *Sp*EgtU_S_P5/*Sp*EgtU_S_P6, with the genomic DNA of IU9985 containing sfGFP DNA sequence as a template^78^. The sfGFP CTD and NTD fragments were linked together by fusion PCR using primer pairs *Sp*EgtU_S_P3/*Sp*EgtU_S_P6 to generate fragment F2. Fragment F2 was ligated to fragment F1 by a Gibson assembly protocol^79^. Expression vectors were amplified in *E. coli* DH5**α** and sequences verified.

The sequence-verified expression vectors were transformed into *E. coli* BL21(DE3) and grown in either LB (*S. pneumoniae*, *E. faecalis*) or an M9 minimal medium (*L. monocytogenes*, *S. aureus*) supplemented with 30 µg/mL kanamycin. 1 mM isopropyl β-*D*−1-thiogalactopyranoside (IPTG) was added to induce protein expression at OD_600_ ≈ 0.8. Following overnight expression at 18 °C, the cells were pelleted by centrifugation. The cell pellet was resuspended in Buffer A (25 mM Tris-HCl, pH 8), 500 mM NaCl, 10% glycerol, 20 mM imidazole) and lysed by sonication on ice. The crude lysate was clarified by centrifugation. 70% ammonium sulfate was applied to precipitate the protein and the pellet was collected by centrifugation. The precipitated pellet was resuspended in Buffer A and the solution subjected to Ni(II) immobilized affinity chromatography using a 5 mL HisTrap FF column (GE Healthcare Life Sciences) with a gradient from 100% buffer A to 100% buffer B (25 mM Tris-HCl, pH 8.0, 500 mM NaCl, 10% glycerol, 500 mM imidazole). The fractions containing the His-tagged SUMO fusion protein were pooled and digested by SUMO protease (20 µg/mL) while dialyzing in buffer A with 2 mM dithiothreitol (DTT) at room temperature. The digested protein fractions were applied to a HisTrap FF column in Buffer A. The flow-through fractions were pooled and concentrated by centrifugation with a 10 kDa cutoff and subjected to size exclusion chromatography on a Superdex-200 column in Buffer C (25 mM Tris-HCl, pH 8.0, 500 mM NaCl, 2 mM EDTA) and monomeric fractions pooled. The concentration of purified protein was measured using the molar extinction coefficients at 280 nm (ε_280_) (Supplementary Table 6). Purified protein fractions were pooled and stored at –80 °C until use.

### Intrinsic tyrosine fluorescence titration analysis

Data were acquired on a PC1 spectrofluorometer with *λ*_ex_ 285 nm (2 mm slit) and the emission intensity recorded through a 305 nm cut-off filter. The ligand was prepared in titration buffer (50 mM HEPES, pH 7.5, 150 mM NaCl, 2 mM EDTA). All proteins were buffer exchanged into the same titration buffer and ligands were titrated into 3 mL 1 µM protein. The titrations were carried out with continuous stirring at 25.0 (±0.1) °C and resulting data corrected for dilution and the inner filter effect and fit to a 1:1 protein:ligand binding model to estimate *K*_*a*_ using DynaFit^80^, assuming a linear relationship of fluorescence signal change to fractional occupancy of EgtUC with ET.

### Isothermal calorimetry titration

ITC experiments were carried out using a MicroCal VP-ITC calorimeter at 25 (±0.1) °C by titrating 20 or 30 µM *Sp*EgtUC or the indicated mutant in the sample chamber in 50 mM HEPES, pH 7.5, 150 mM NaCl, 2 mM EDTA with the indicated ligand (ET, *L*-hercynine, *L*-histidine or GB) in the syringe in same buffer. For the ET titration, the ligand concentration in the syringe was typically 375 μM with 30 μM protein in the sample chamber. For other ligands, the ligand concentration was 600 μM, and 20 μM protein in chamber. The raw ITC data were integrated, concentration normalized, and plotted as heat versus ligand/protein ratio using Origin. All data were fit to a single site binding model included in the data analysis package provided by MicroCal.

### Sypro Orange differential scanning fluorimetry

Sypro Orange thermal denaturation assays were carried out for each mutant in triplicate using a 96-well plate StepOne Plus RT-PCR machine (Applied Biosystems). Each well contained 20 μL solution with 10 μM protein, 10× Sypro Orange dye, 50 mM HEPES, pH 7.5, 150 mM NaCl, 2 mM EDTA. 100 μM ET was used for wells containing ET. Hercynine concentrations ranged from 10 μM to 1000 μM. The temperature was increased from 25 °C to 95 °C at a ramp rate of 1.5 °C per minute. Apparent melting temperatures (*T*_m_) were determined from the maximum of the first derivative of the fluorescence intensity curve^81^.

### *Sp*EgtUC crystallography and data analysis

The purified protein was buffer exchanged into crystallography buffer, 50 mM Tris-HCl, 150 mM NaCl, 2 mM EDTA pH 7.5. A 3-fold excess of ET was added to the purified protein and excess ligand removed by chromatography on a HiLoad 16/600 Superdex 200 size exclusion column (Cytiva). The main peak corresponding to monomeric *Sp*EgtUC was pooled and concentrated for protein crystallography screening. *Sp*EgtUC_CTT_–ET (15 mg/mL) crystals grew in sodium citrate, pH 5.6, 0.2 M potassium sodium tartrate and 1.8–2.0 M ammonium sulfate at 20 °C using the hanging-drop vapor-diffusion method. *Sp*EgtUC-ET (15 mg/mL) crystals grew in 1.6 M sodium citrate, pH 6.5, at 20 °C using the hanging-drop vapor-diffusion method. Crystals were harvested, cryo-protected in a reservoir solution supplemented with 25% glycerol and flash-frozen in liquid nitrogen. Diffraction data were collected at 100 K at the Beamline station 4.2.2 at the Advanced Light Source (Berkeley National Laboratory, CA) and were initially indexed, integrated, and scaled using XDS^82^. Molecular replacement was used to estimate phases using PHASER and PDB code 4Z7E^38^ as search model. Successive cycles of automatic building in Autobuild (PHENIX) and manual building in Coot, as well as refinement (PHENIX Refine) led to complete models^83^. MolProbity software^84^ was used to assess the geometric quality of the models, and Pymol was used to generate molecular images. Data collection and refinement statistics are indicated (Supplementary Table 1).

### Structure modeling

Apo *Sp*EgtUC was modeled using ColabFold on https://colab.research.google.com/github/sokrypton/ColabFold/blob/main/beta/AlphaFold2_advanced.ipynb with AlphaFold2 downloaded from https://github.com/deepmind/alphafold on Sep 2, 2021. *Sp*EgtUC-GFP and the *Sp*EgtUBC dimer were modeled using colabfold version 1.3, using localcolabfold downloaded from https://github.com/YoshitakaMo/localcolabfold and installed on 4 July 2022 with default parameters. The GFP chromophore was added to the image using PDB 7S7V and Pymol. The rank 1 model of *Sp*EgtUBC was submitted to https://consurf.tau.ac.il/consurf-old.php with a custom multiple sequence alignment from the sequence similarity network analysis to color by sequence conservation.

### Position-induced quenching of bimane fluorescence

F277W/L374C *Sp*EgtUC was prepared as described above except that buffer for protein purification was degassed and 2 mM TCEP was added to all purification buffers. The purified protein was then buffer exchanged into degassed labeling buffer, 200 mM sodium phosphate, pH 7.4 without reducing reagent. A mBBr stock solution was prepared in DMSO at 20 mM and stored at –80 °C until used. 20 µM protein was mixed with a 30-fold molar excess of mBBr in labeling buffer at 37 °C for 1 h and excess mBBr removed by eight rounds of washing through a 10 kDa cut-off centrifugation filter with labeling buffer. The concentration of the labeled protein was measured by absorption at 280 nm using the molar extinction coefficient shown (Supplementary Table 6). The conjugated bimane concentration was measured using an ε_380_ of 5000 M^−1^ cm^−1^. Data were acquired in 50 mM HEPES, pH 7.5, 150 mM NaCl, 2 mM EDTA on a PC1 spectrofluorometer with λ_ex_ 380 nm (2 mm slit) and the emission intensity recorded through a 480 nm cut-off filter. Ligands were prepared in this buffer with the protein buffer exchanged into the same buffer. Various ligands were titrated into 3 mL 1 µM protein up to 5 µM total ligand, with continuous stirring at 25.0 (±0.1) °C. All titration data were fit to a 1:1 protein:ligand binding model to estimate *K*_a_ using DynaFit^80^. The emission spectrum was acquired from 400 nm to 650 nm before and after the titration, with the initial emission intensity at 480 nm normalized to 1 and that at 650 nm normalized to 0.

### NMR backbone assignments

Uniformly ^15^N, ^13^C, ^2^H-labeled *Sp*EgtUC was expressed in *E. coli* BL21 (DE3) cells in M9 minimal medium containing 1 kg D_2_O, as well as 1.0 g of ^15^NH_4_Cl and 2 g ^13^C_6_,^2^H-glucose as the sole nitrogen and carbon sources, respectively. Uniformly ^15^N-labeled protein was expressed in *E. coli* BL21 (DE3) cells in M9 minimal medium containing 1.0 g of ^15^NH_4_Cl as the sole nitrogen source. Further expression, isolation, and purification of these isotope-labeled proteins was performed as described above for unlabeled protein. To facilitate exchange of deuterated amides back to protons, the purified protein was incubated with 2.5 M guanidinium-HCl and 5 mM EDTA for 3 h, then dialyzed into NMR buffer (10 mM sodium phosphate, pH 7.0, 150 mM NaCl). ^15^N TROSY spectra on samples labeled with only ^15^N were used to confirm nearly complete back-exchange of the deuterated sample. NMR spectra were recorded at 35 °C on a 600 MHz Bruker Avance Neo spectrometer equipped with a cryogenic probe in the METACyt Biomolecular NMR Laboratory at Indiana University, Bloomington.

NMR samples for backbone assignment contained 0.75 mM ^15^N, ^13^C, ^2^H -labeled protein, with or without 0.75 mM ET, in 10 mM sodium phosphate pH 7.0, 150 mM NaCl, and 10% v/v D_2_O, with 0.3 mM 2,2-dimethyl-2-silapentanesulfonic acid (DSS) as an internal reference. Backbone chemical shifts were assigned for each state using TROSY versions of the following standard triple-resonance experiments: HNCACB, HNCOCACB, HNCA, HNCOCA, HNCO, and HNCACO, using non-uniform sampling with Poisson gap schedules. Data were collected using Topspin 4.1.3 (Bruker) and processed using NMRPipe and istHMS, and analyzed using CARA and Sparky, all on NMRbox^85^ as described^74^. TALOS-N was used for chemical shift-based secondary structure predictions^86^. Chemical shift perturbations (CSP) of the backbone upon ligand binding were calculated using ^1^H and ^15^N chemical shifts with Δ*δ* = ((Δδ*H*)^2^ + 0.2(ΔδN)2)^1/2^. Chemical shift perturbations upon interaction with *L*-hercynine were monitored using 0.2 mM ^15^N EgtUC and concentrations ranging up to 2 mM. A total of 30 mM GB was titrated into 0.15 mM ^15^N-labeled EgtUC.

### ^15^N spin relaxation experiments

NMR samples for relaxation experiments contained 0.75 mM ^15^N-labeled protein, with or without 0.75 mM ET, in 10 mM sodium phosphate pH 7.0, 150 mM NaCl, and 10% v/v D_2_O, with 0.3 mM 2,2-dimethyl-2-silapentanesulfonic acid (DSS) as an internal reference. The ^15^N spin relaxation rates *R*_1_ and *R*_2_, and ^1^H-^15^N heteronuclear NOE (hNOE) values were measured using TROSY pulse sequences. The relaxation delays used were 0.05, 0.20, 0.50, 0.80, 1.2, 1.6, 2.0, and 2.5 s for *R*_1_ and 0.017, 0.034, 0.051, 0.068, 0.085, 0.102, 0.119, 0.136, 0.170, and 0.204 s for *R*_2_. Residue-specific *R*_1_ and *R*_2_ values were obtained from fits of peak intensities vs. relaxation time to a single exponential decay function, while hNOE ratios were ascertained directly from intensities in experiments recorded with (2 s relaxation delay followed by 3 s saturation) and without saturation (relaxation delay of 5 s). Errors in hNOE values were calculated by propagating the error from the signal to noise. Hydrogen atoms were added to the crystal structure coordinates for ET-bound WT EgtUC and to the AlphaFold2 model of the apo state using the PDB utilities at http://spin.niddk.nih.gov/bax/nmrserver/pdbutil in order to obtain structure-based predictions for relaxation rates using HYDRONMR^52^. A value for the atomic radius element of 3.8 Å, the known viscosity for water at 35 °C, and CSA of –120 ppm were used for this calculation.

### Ligand specificity analysis using the *Sp*EgtUC-GFP titration assay

To measure the ET binding affinity with *Sp*EgtUC-GFP, the fluorescence change upon ET titration was acquired on a PC1 spectrofluorometer with excitation at 485 nm (2 mm slit) and total emission recorded through a 510 nm cut-off filter in titration buffer (50 mM HEPES, pH 7.5, 150 mM NaCl, 2 mM EDTA) with 2 mM TCEP. ET was titrated into 3 mL 1 µM protein in the same buffer until saturation of the protein was reached. The titration was done with continuous stirring at 25.0 (±0.1) °C and the resulting data fit to a 1:1 protein:ligand binding model to estimate *K*_a_ using DynaFit^80^. The emission spectrum from 400 nm to 650 nm was measured before and after the titration. The initial emission intensity at 510 nm was set to 1, emission intensity at 650 nm set to 0.

To analyze the ligand specificity of *Sp*EgtUC-GFP, triplicate 1 μM protein samples were mixed with 0, 1.0, 10, and 100 μM of the indicated ligand in 100 µL titration buffer (50 mM HEPES, pH 7.5, 150 mM NaCl, 2 mM EDTA) with 2 mM TCEP added in a 96-well plate at 25 °C. Ligands include *L*-ergothioneine (ET), *L*-hercynine (HER), *L*-histidine (HIS), glycine betaine (GB), proline betaine (PB), choline (CHO), ectoine (ECO), *L*-carnitine (CAR) and dimethylsufoniopropionate (DMSP). Fluorescence was obtained by excitation at 485 nm and emission at 510 nm. After the fluorescence intensity was determined, ET was added into samples to 1.0 µM with 100 μM of the indicated ligand, with the fluorescence intensity of those samples measured again. The change in fluorescence intensity, Δ*F*, between ET-added samples (Fs) and ET-free samples (Fo) were normalized to the ratio R defined as (|Fo-Fs|)/Fo.

### EFI-GNN analysis

A sequence similarity network was generated using the sequence BLAST option with *Sp*EgtUBC as the query sequence of the UniProt database using the default UniProt BLAST E-value of 5 using the Enzyme Function Institute–Enzyme Similarity Tool (EFI-EST; https://efi.igb.illinois.edu/efi-est/)^55^. All of the resulting sequences belonged to the pfam protein family PF04069 and were retrieved in December 2021 using the UniRef90 option. This option takes sequences that share ≥90% sequence identity over 80% of the sequence length, groups them together and represents them by a sequence known as the cluster ID. The resulting sequence file was subjected to SSN analysis using an alignment score of 120 and a minimum and maximum sequence length of 250 and 650 residues in an effort to eliminate truncation artifacts. The resulting SSN was colored and found to contain 19,991 metanodes and 57,649 unique accession IDs that segregate into 2044 non-singleton clusters and 2458 singletons and displayed as a repnode (representative node) 60 file (sequences with 60% identity over 80% of the sequences represented by a single node), analyzed and annotated using Cytoscape. Multiple sequence alignments from each SSN cluster were trimmed for easier visualization using the tool CIAlign^87^ to remove insertions found in fewer than half of the sequences and to crop any poorly aligned termini of sequences. The trimmed multiple sequence alignments were then visualized using WebLogo 3.

### Statistical analysis methods

The number of biological or independent replicates (*n*) is indicated for each experiment and whenever possible all experimental data points are shown along with the standard deviation. No statistical method was used to predetermine the sample size.

### Reporting summary

Further information on research design is available in the Nature Portfolio Reporting Summary linked to this article.

## Supplementary information

Supplementary Information

Reporting Summary

## Data Availability

The data that support this study are available from the corresponding author upon request. The crystallographic structures have been deposited in the Protein Data Bank under accession codes 7TXL (*Sp*EgtUC structure) and 7TXK (*Sp*EgtUC_CTT_ structure). NMR data are available from the BMRB under accession codes 51423 (apo-*Sp*EgtUC) and 51424 (ET-bound *Sp*EgtUC). AlphaFold2 models are available at https://modelarchive.org/ under the accession codes ma-xwg27 (apo-*Sp*EgtUC), ma-8paa8 (*Sp*EgtUC-GFP), and ma-42n23 (*Sp*EgtUB dimer with a single docked EgtUC). Source data are provided with this paper.

## Source data

Source Data
