# Supplementary material for: Discovery and structure of a widespread bacterial ABC transporter specific for ergothioneine
Source: Nat Commun. 2022 Dec 8;13:7586. doi: 10.1038/s41467-022-35277-3 (PMC9732360; doi:10.1038/s41467-022-35277-3)
Supplement: Supplementary file 1 — Supplementary Information [file 41467_2022_35277_MOESM1_ESM.pdf]

Supplementary Information

Discovery and structure of a widespread bacterial ABC transporter specific for ergothioneine

Yifan Zhang[1,2], Giovanni Gonzalez-Gutierrez[2], Katherine A. Legg[1], Brenna J.C. Walsh[1,3], Cristian M. Pis Diez[4,5], Katherine A. Edmonds[1,¶], and David P. Giedroc[1,2,¶,*]

[1]Department of Chemistry, Indiana University, Bloomington, IN USA
[2]Department of Molecular and Cellular Biochemistry, Indiana University, Bloomington, IN USA
[3]Present address: Research and Exploratory Development Department, The Johns Hopkins University Applied Physics Laboratory, 11100 Johns Hopkins Road, Laurel, Maryland 20723, USA
[4]Fundación Instituto Leloir, Av. Patricias Argentinas 435, Buenos Aires C1405BWE, Argentina
[5]Present address: Department of Chemistry, New York University, 100 Washington Square E, New York, NY 10003 USA

[¶]These authors share senior authorship.
*Correspondence giedroc@indiana.edu

This file contains **Supplementary Figures 1-20**, **Supplemental Tables 1-6** and Supplementary References

TABLE OF CONTENTS

Item	Page
**Supplementary Figure 1.** ET tautomerization equilibrium and chemical structures and exact masses of HPE-IAM-derivatized thiols used in this work…………………...	S4
**Supplementary Figure 2.** Mass spectrometry of light (H_4) and heavy (D_4) HPE-IAM-capped thiol standards…………………………………………………………………	S5
**Supplementary Figure 3.** Mass spectrometry of light (H_4) and heavy (D_4) HPE-IAM-capped thiols obtained from *S. pneumoniae* D39 cell lysates grown on BHI…………	S6
**Supplementary Figure 4.** Mass spectrometry of light (H_4) and heavy (D_4) HPE-IAM-capped thiols obtained from *S. pneumoniae* D39 cell lysates grown on a chemically defined growth medium (CDM) to which exogenous ET was added…………………...	S7
**Supplementary Figure 5.** Estimated intracellular concentrations of LMW thiols	S8
**Supplementary Figure 6.** Predicted transmembrane topology of EgtUBC……………	S9
**Supplementary Figure 7.** Size exclusion chromatography of wild-type (WT) and selected mutant *Sp*EgtUCs………………………………………………………………	S10
**Supplementary Figure 8.** Differential scanning fluorimetry (DSF) of *Sp*EgtUC WT and mutants, acquired with SYPRO orange………………………………………………	S11
**Supplementary Figure 9**. Replacement and truncation of the C-terminal five residues of *Sp*EgtUC (GLLKK) with VC has a minimal effect on the structure and no impact on ET-binding affinity………………………………………………………………………	S12
**Supplementary Figure 10.** Comparison of EgtU binding pocket with other subcluster F-III proteins………………………………………………………………………………..	S13
**Supplementary Figure 11**. Conformational changes and dynamics of *Sp*EgtUC in solution…………………………………………………………………………………..	S14
**Supplementary Figure 12.** Backbone $^1H,^{15}N$ assignments of apo *Sp*EgtUC…………	S15
**Supplementary Figure 13.** Backbone $^1H,^{15}N$ assignments of ET-bound *Sp*EgtUC….	S16
**Supplementary Figure 14.** Chemical structures of QAC ligands used in this work…. and representative ITC titrations of wild-type *Sp*EgtUC with non-cognate ligands……	S17
**Supplementary Figure 15.** NMR-monitored titrations of SpEgtUC with non-cognate and weakly binding ligands *L*-hercynine and glycine-betaine……………………………	S18
**Supplementary Figure 16**. Sequence similarity network (SSN) of solute binding proteins (domains) most closely related to *Sp*EgtUC…………………………………….	S19
**Supplementary Figure 17.** Network connectivity analysis……………………………..	S20
**Supplementary Figure 18.** The biological range determined for the EgtU subcluster of cluster 2…………………………………………………………………………………	S21
**Supplementary Figure 19.** Sequence conservation maps of SSN cluster 2 subclusters…..………………………………………………………………………………	S22
**Supplementary Figure 20.** ET-binding properties of candidate EgtU homologs from other firmicutes. ………………………………………………………………………	S23
**Supplementary Table 1.** Data collection and refinement statistics. ……………….....	S24
**Supplementary Table 2.** List of candidate C-H•••S hydrogen bonding interactions in the EgtUC-ET complex……………………………………………………………………	S25
**Supplementary Table 3.** List of water molecules and associated B-factors in the structures of *Sp*EgtUC$_{CTT}$ and *Sp*EgtUC………………………………………………...	S26
**Supplementary Table 4.** List of *Streptococcus pneumoniae* D39 strains in this work	S27
**Supplementary Table 5.** Primers used in this study…………………………………….	S27
**Supplementary Table 6.** Molar extinction coefficients of purified proteins at 280 nm (ε_{280}) used in this work………………………………………………………………	S29

Supplementary References……………………………………………………………............. S29

SUPPLEMENTARY FIGURES

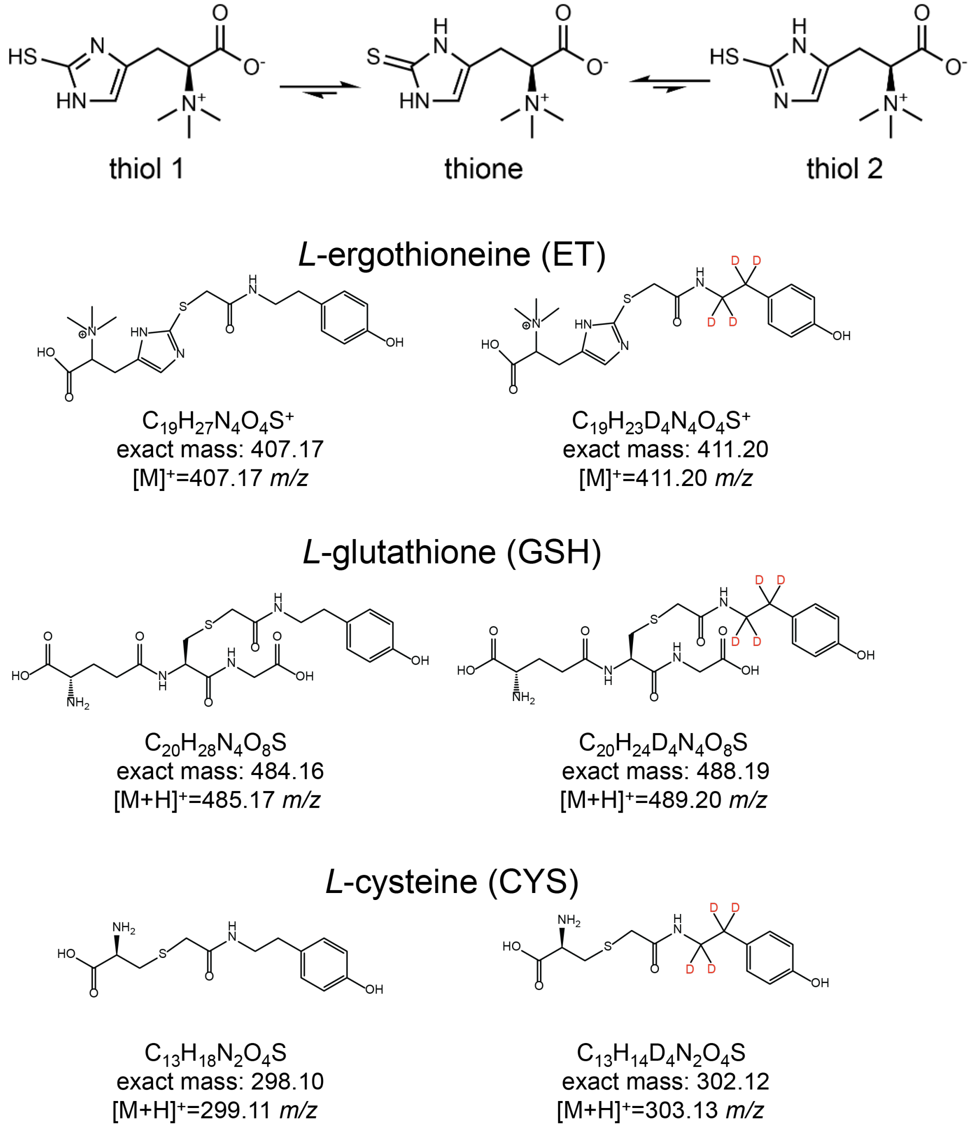

Supplementary Figure 1. ET tautomerization equilibrium and chemical structures and exact masses of HPE-IAM-derivatized thiols used in this work. The light (H_4; *left*) and heavy (*D_4*; *right*) HPE-IAM adducts of *L*-ergothioneine (*top*), *L*-glutathione (*middle*) and *L*-cysteine (*bottom*).

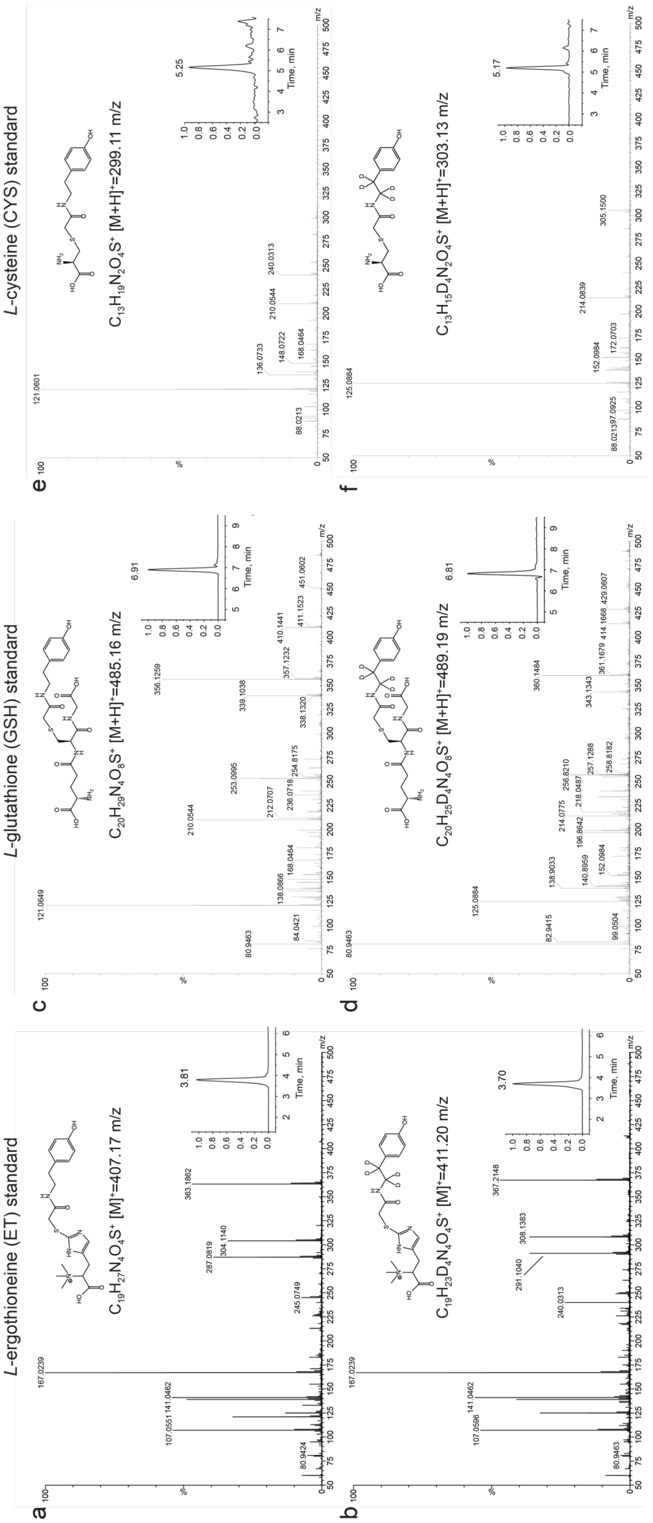

Supplementary Figure 2. Mass spectrometry of light (H_4) and heavy (*D_4*) HPE-IAM-capped thiol standards. LC-MS (MS1) (*insets*) and LC-MS/MS (normalized ion count) of authentic HPE-IAM capped LMW thiols are shown. **a** and **b**, *L*-ergothioneine; **c** and **d**, *L*-glutathione; **e** and **f**, *L*-cysteine. Structure of the compound and expected masses used to query the TIC are shown.

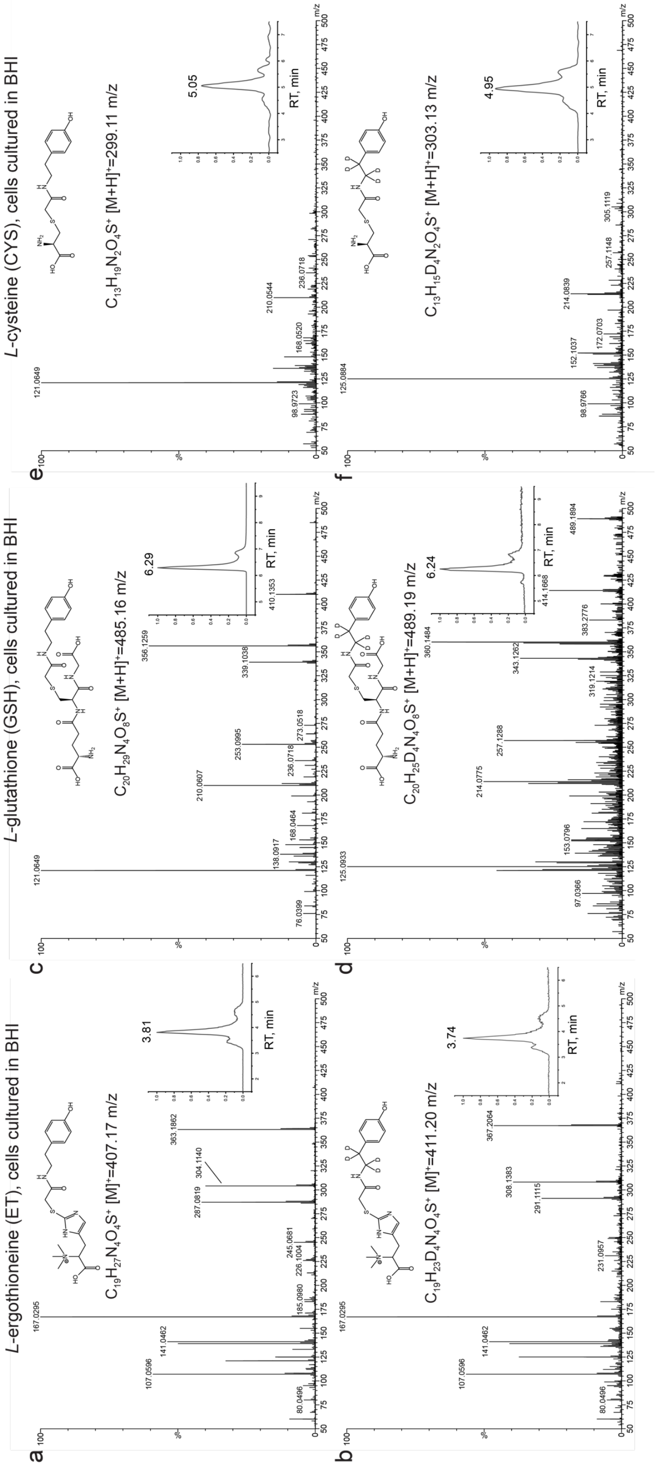

Supplementary Figure 3. Mass spectrometry of light (H_4) and heavy (*D_4*) HPE-IAM-capped thiols obtained from *S. pneumoniae* D39 cell lysates grown on BHI. LC-MS (MS1) (*insets*) and LC-MS/MS (normalized ion count) of HPE-IAM capped LMW thiols are shown. **a** and **b**, *L*-ergothioneine; **c** and **d**, *L*-glutathione; **e** and **f**, *L*-cysteine. Structure of the compound and expected masses used to query the TIC are shown.

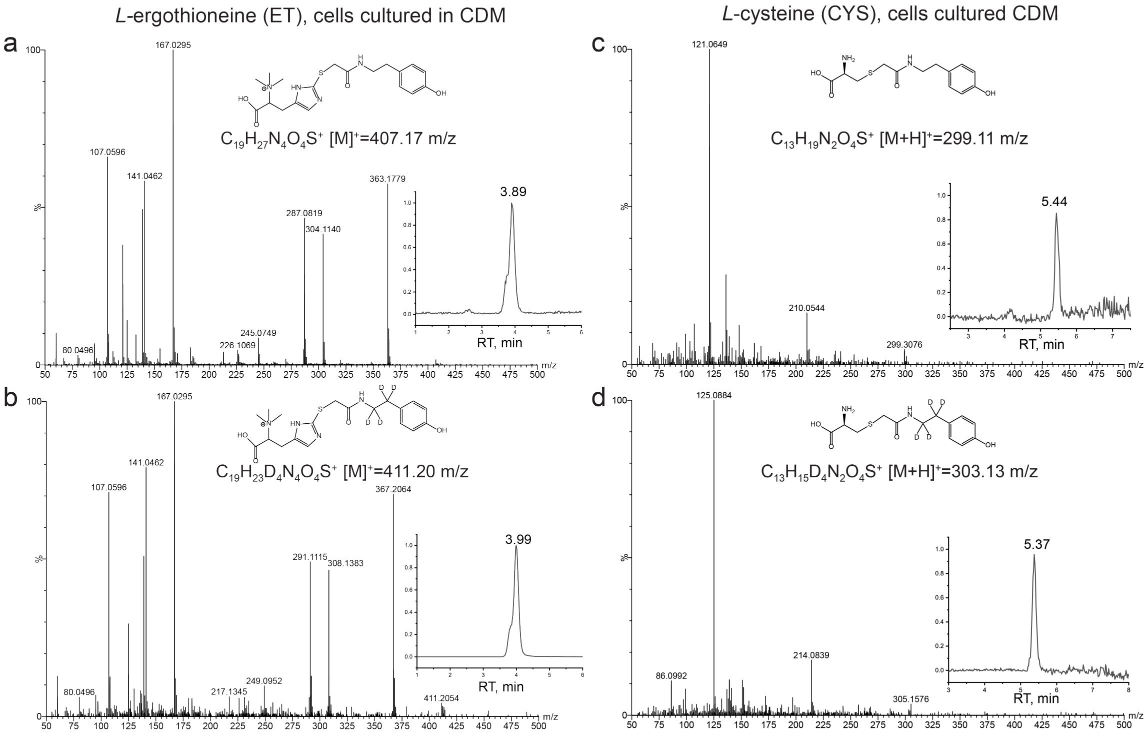

Supplementary Figure 4. Mass spectrometry of light (H_4) and heavy (D_4) HPE-IAM-capped thiols obtained from *S. pneumoniae* D39 cell lysates grown on a chemically defined growth medium (CDM) to which exogenous ET was added. LC-MS (MS1) (*insets*) and LC-MS/MS (normalized ion count) of HPE-IAM capped LMW thiols are shown. **a** and **b**, *L*-ergothioneine; **c** and **d**, *L*-cysteine. Structure of the compound and expected masses used to query the TIC are shown. Note that there is no detectable *L*-glutathione in these lysates.

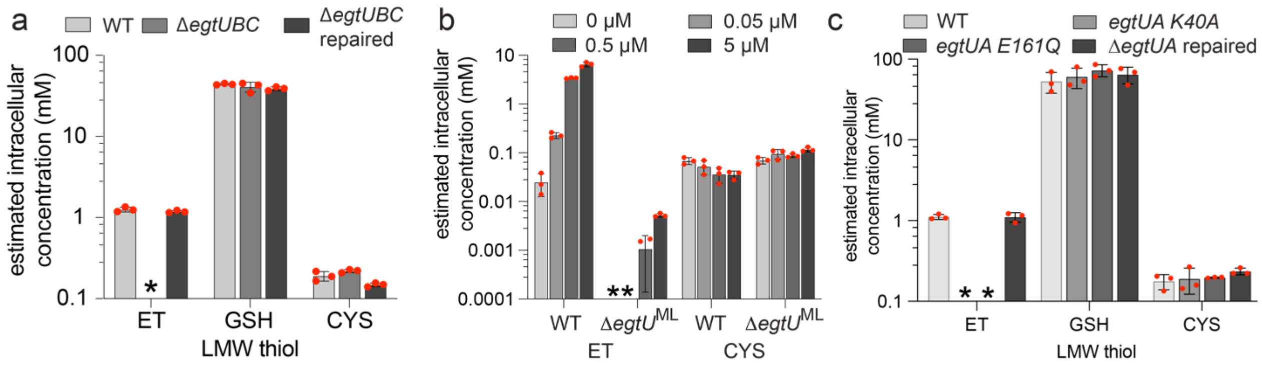

Supplementary Figure 5. Estimated intracellular concentrations of LMW thiols. These estimates were calculated using a value of 5.5×10^8 cells per unit OD_{620} and intracellular volume of 6×10^{-16} L per cell.[1,2] We note that these estimates are approximate and assume an identical cell-associated volume and average chain length of two cells for all pneumococcal strains independent of the growth media, and does not distinguish between cytoplasmic and externally bound ET. **a**, Estimated intracellular concentration of ET, GSH, and CYS found in the indicated strains of *S. pneumoniae* D39 grown in BHI in biological triplicate. *, not detected (≤0.001 mM). **b**, Estimated concentration of ET and CYS in the indicated strains of *S. pneumoniae* D39 grown in chemically-defined medium (CDM) supplemented with indicated concentration of ET in biological triplicate. *, below the limit of detection (≤0.1 μM). **c**, Estimated intracellular concentration of ET, GSH, and CYS found in the indicated strains of *S. pneumoniae* D39 grown in BHI in biological triplicate. *, not detected (≤0.001 mM). Each data point is shown as the mean and standard deviation of three independent replicates, with individual measurements shown as red circles. Source data are available as a Source Data file.

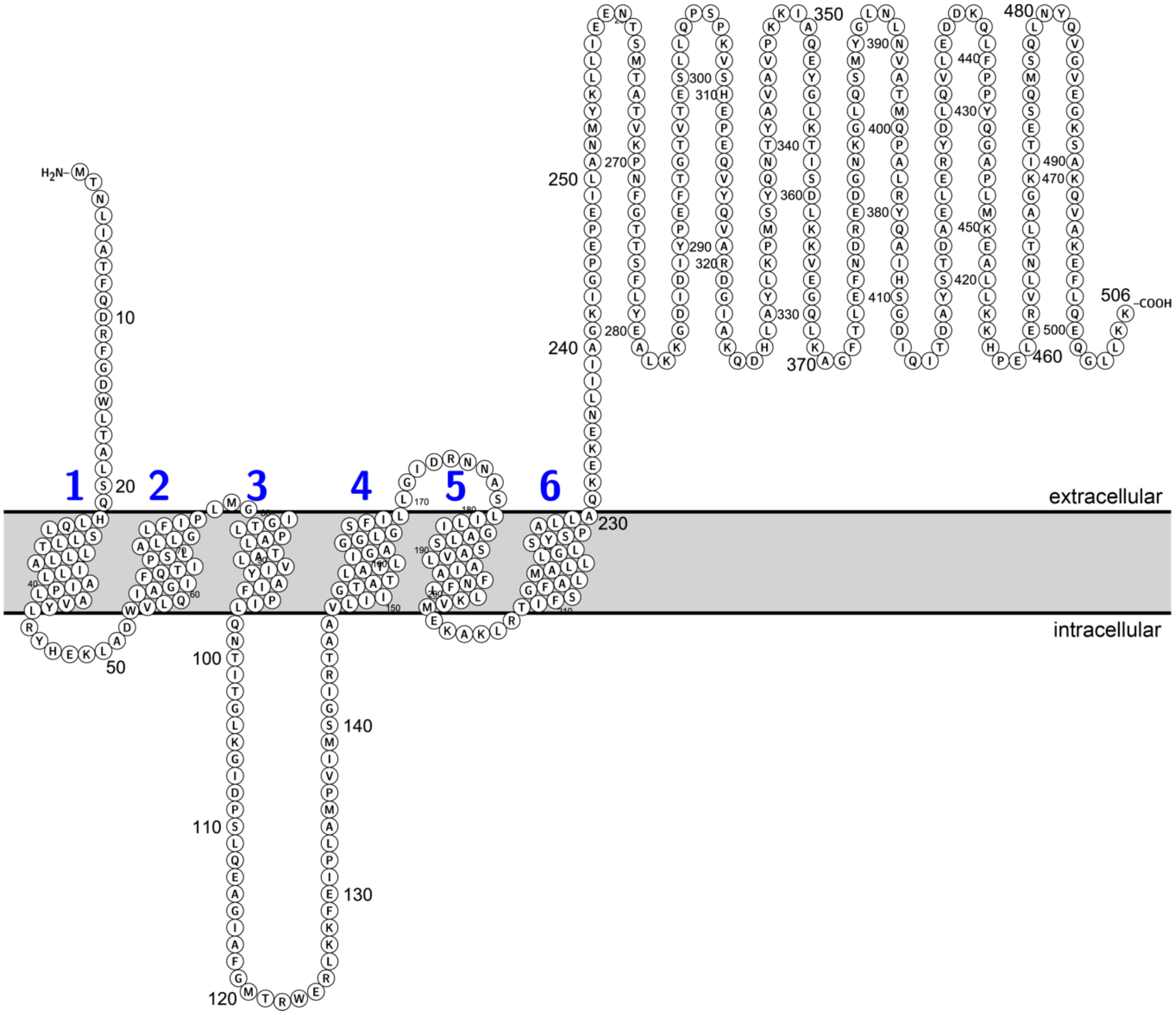

Supplementary Figure 6. Predicted transmembrane topology of EgtUBC. EgtUBC is predicted to contain a transmembrane domain consisting of six transmembrane helices between residues 20 and 230, followed by an extracellular domain between residues 230 and 506. These predictions were generated by TMHMM2.0[3] and displayed by Protter[4].

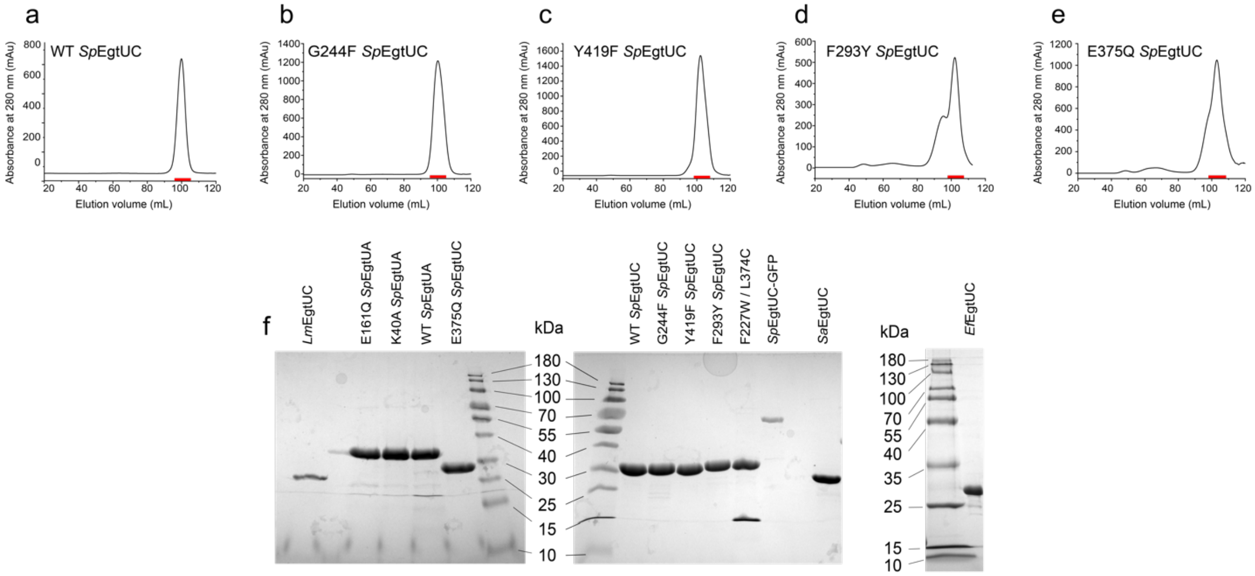

Supplementary Figure 7. Homogeneity analysis of EgtUC preparations. a-e, Size exclusion chromatography purification of wild-type (WT) and selected mutant *Sp*EgtUCs. The indicated fractions defined by the red lines were pooled and used for biochemical analysis. **f,** SDS-PAGE analysis of these same EgtUC preparations used in this work. 7 μg of protein was loaded in each lane. The bands shown on these SDS-PAGE gels are representative of at least two replicate gels.

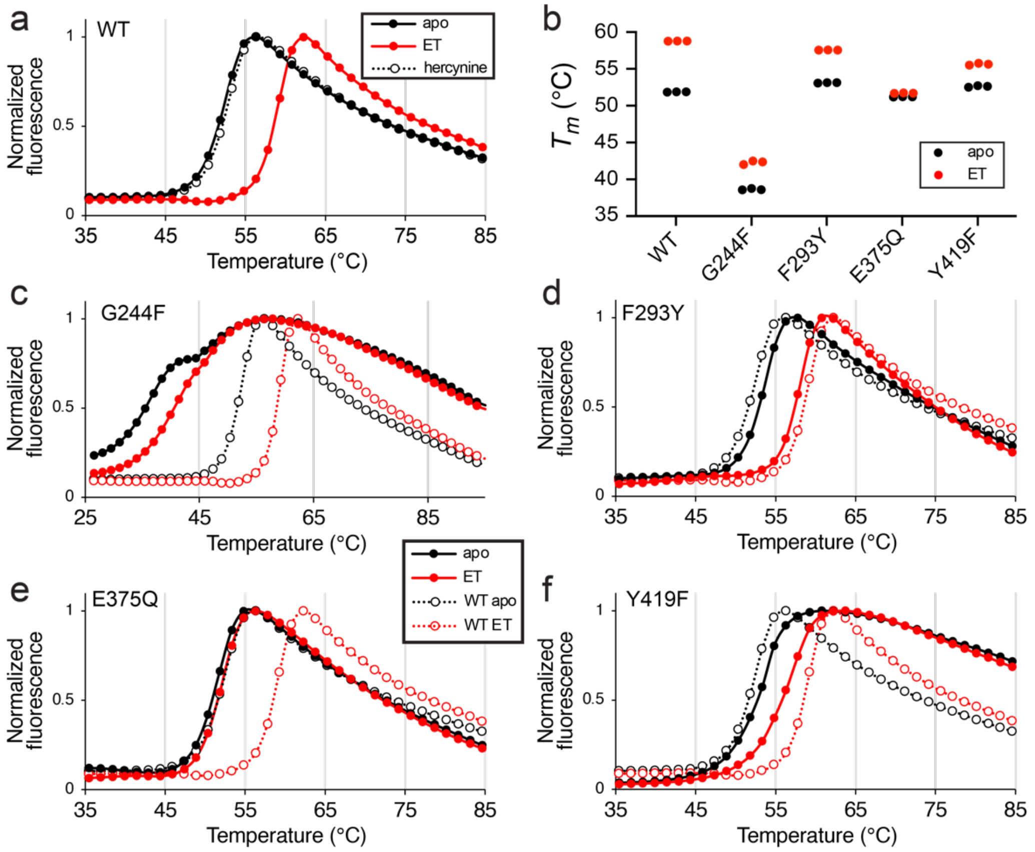

Supplementary Figure 8. Differential scanning fluorimetry (DSF) of *Sp*EgtUC WT and mutants, acquired with SYPRO orange. a, Representative DSF scans of WT *Sp*EgtUC, in the absence of ligand (*black circles*), in the presence of 10-fold excess ET (*red circles*), and in the presence of 100-fold excess hercynine (*open circles*). **b,** Fitted melting temperatures of WT *Sp*EgtUC and each mutant, in the absence (*black circles*) and presence (*red circles*) of 10-fold excess ET, as measured in three independent experiments. **c-f,** Representative DSF scans of the indicated mutant *Sp*EgtUCs, in the presence (*red*) and absence (*black*) of 10-fold excess ET, with the corresponding curves for WT shown with dashed lines and open circles for comparison. All DSF scans were measured in triplicate. Source data are available as a Source Data file.

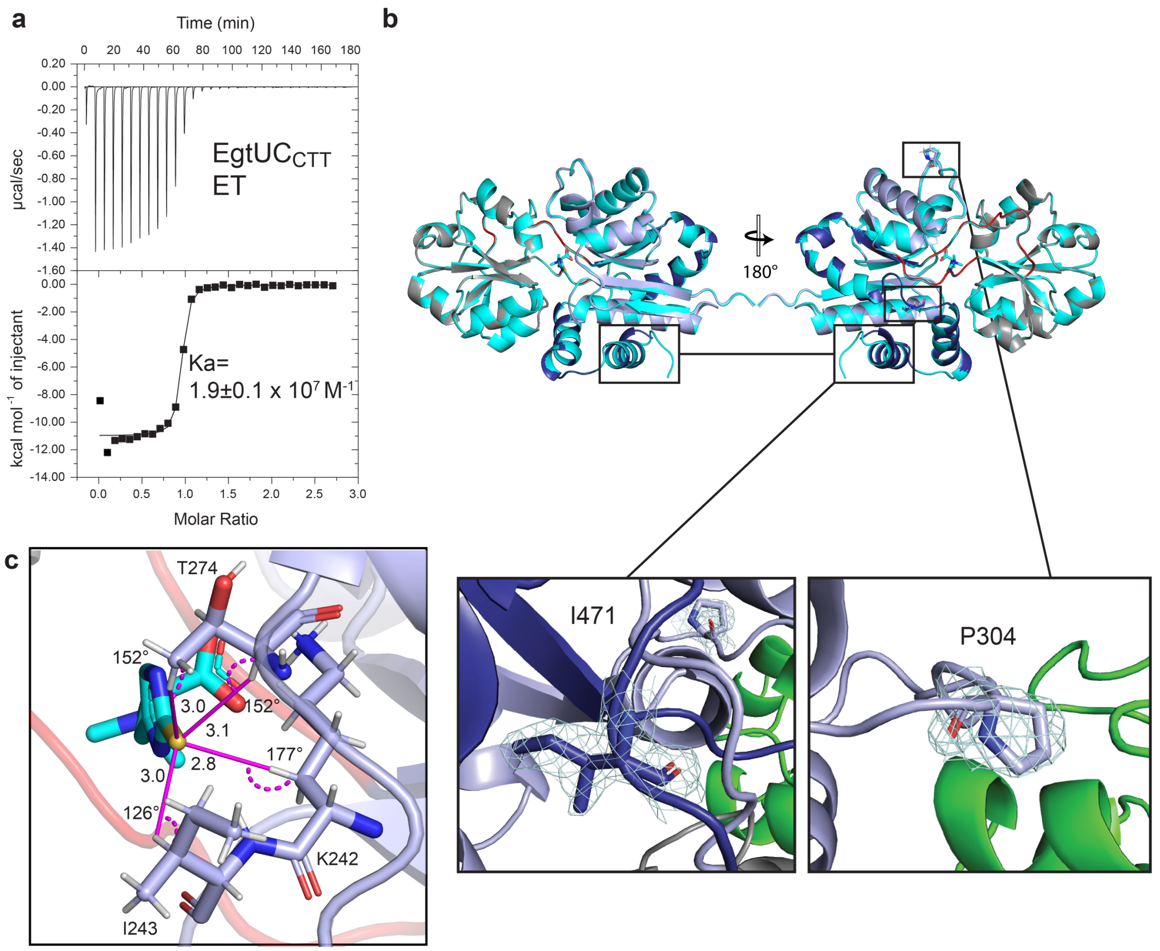

Supplementary Figure 9. Replacement and truncation of the C-terminal five residues of SpEgtUC (GLLKK) with VC has a minimal effect on the structure and no impact on ET-binding affinity. a, Isothermal titration calorimetry of the C-terminally truncated *Sp*EgtUC, designated *Sp*EgtUC$_{CTT}$, titrated with ET. The continuous line through the EgtUC$_{CTT}$ binding data represents a fit to a 1:1 binding model (see Table 1, main text for parameters). The dataset shown is representative of two independent replicates. **b**, Global alignment of the crystal structures of ET-bound *Sp*EgtUC (*cyan*, PDB code: 7TXK) and *Sp*EgtUC$_{CTT}$ (*gray, light blue, and dark blue,* PDB code:7TXL) in cartoon representation (see Supplementary Table 1 for structure statistics). ET is shown as cyan sticks. The structure of the C-terminus is boxed. The expanded regions of the structure shown in the boxes (*top*) highlight an unusual rotamer for I471 side chain and the *cis* conformation of P304 side chain, which is supported by NMR chemical shifts. The other chain in the asymmetric unit is depicted in green. **c**, ET binding pocket of *Sp*EgtUC$_{CTT}$ with C-H•••S hydrogen bonds highlighted in magenta, with H-S distances shown in angstroms.

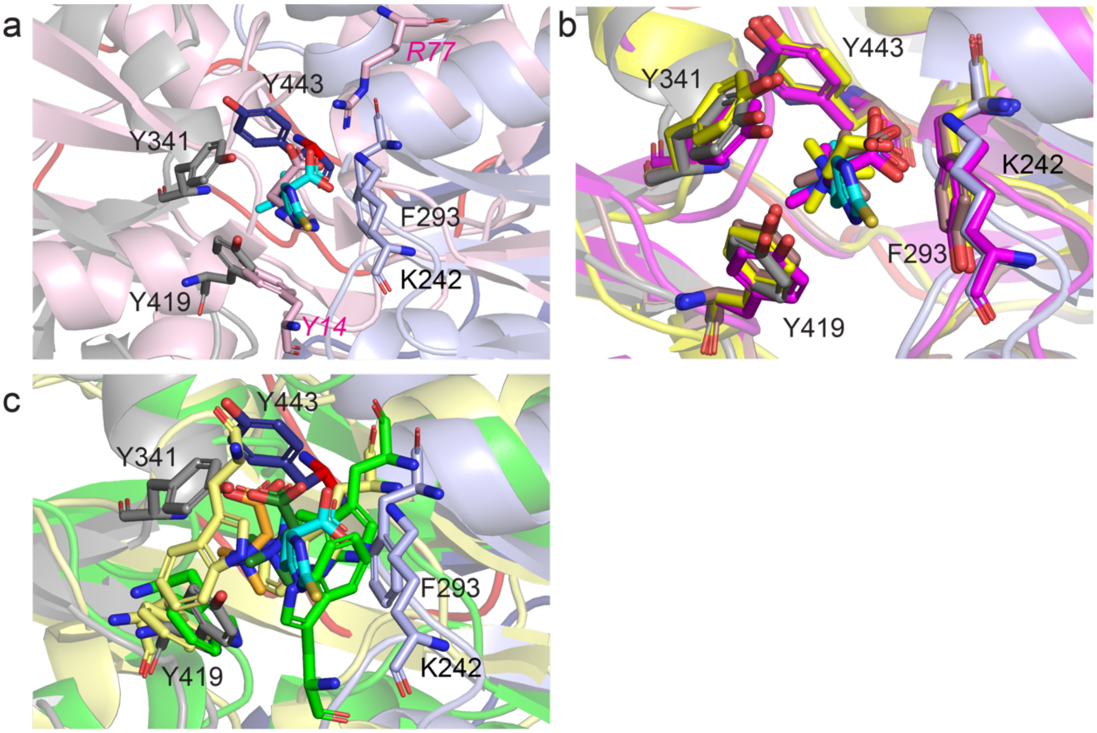

Supplementary Figure 10. Comparison of EgtU binding pocket with other subcluster F-III proteins. a, Overlay of ET in EgtU binding pocket (*blue/gray/cyan*) with histidine in HisJ (*pink*, PDB 1HSL). **b,** Overlay of ET in EgtU binding pocket (*blue/gray/cyan*) with glycine betaine in *Af*ProX (*magenta*, PDB 1SW2), glycine betaine in *Bs*OpuCC (*brown*, PDB 3PPP), and choline in BsOpuBC (*yellow*, PDB 3R6U). **c,** Overlay of ET in EgtU binding pocket (*blue/gray/cyan*) with glycine betaine in *Ll*OpuAC (*green/forest*, PDB 3L6H), glycine betaine in *Ec*ProX (*yellow/gold*, PDB 1R9L)

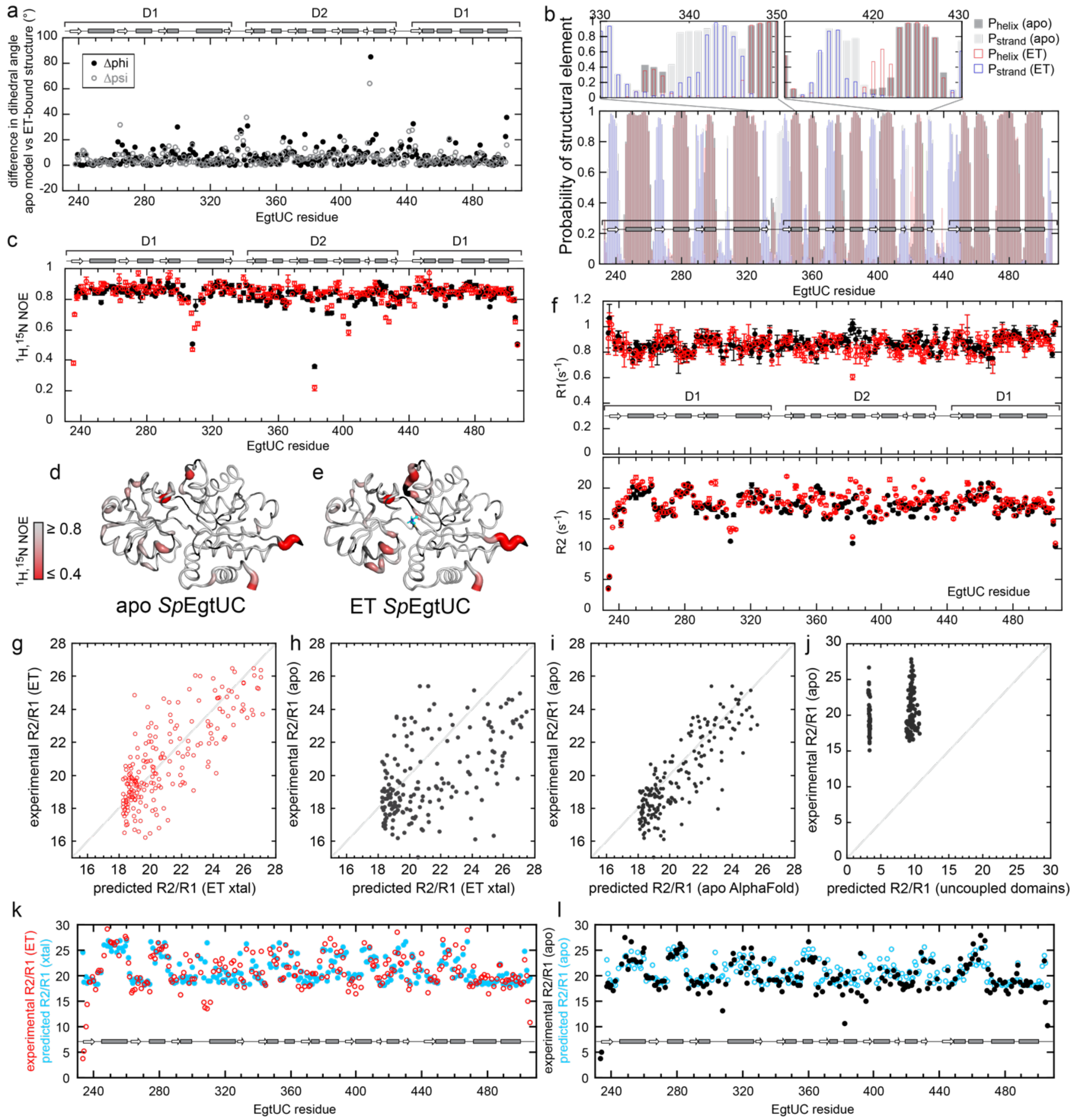

Supplementary Figure 11. Conformational changes and dynamics of *Sp*EgtUC in solution. **a,** Differences in backbone dihedral angles phi and psi between the AlphaFold2 model of apo EgtUC and the crystal structure of ET-bound EgtUC, for each residue predict minimal conformational changes, limited to linkers and loops. **b,** Chemical shift-based secondary structure predictions for EgtUC in the apo and ET-bound states in solution shows extended β strands near the hinge in the apo state. **c,** Steady-state heteronuclear $^{15}N[^{1}H]$ NOE reporting on sub-nanosecond amide bond vector motions of for *Sp*EgtUC residues 235-506 in the apo (black circles) and ET-bound *Sp*EgtUC (red circles)states. **d,** Heteronuclear $^{15}N[^{1}H]$ NOE values for apo *Sp*EgtUC painted onto the WT crystal structure, with more mobile residues represented as thicker tubes and colored darker red. Residues omitted due to resonance overlap or lack of assignment (prolines) are shaded black. Heteronuclear NOE data were recorded as one replicate, with error bars indicating the uncertainty derived from spectral noise. **e,** Heteronuclear $^{15}N[^{1}H]$ NOE values

for ET-bound *Sp*EgtUC painted as in **d**, with unassigned residues shaded black. **f,** Backbone ^{15}N longitudinal (top) and transverse (bottom) spin relaxation rates of *Sp*EgtUC in the apo (black circles) and ET-bound (red circles) forms. Longitudinal and transverse relaxation data were each recorded as one replicate, with error bars indicating the error in the exponential fit. **g.** Comparison between experimental and HYDRONMR calculated values of R2/R1 for ET-bound EgtUC based on the WT crystal structure. **h-j**. Comparison between experimental and HYDRONMR calculated values of R2/R1 for apo EgtUC based on three different models: **h.** an apo structure identical to the ET-bound crystal structure, **i.** an AlphaFold2 model of the open state, and **j.** a model of independently tumbling middle and terminal domains with an infinitely flexible linker. **k.** Experimental (red) and predicted (blue) R2/R1 values for ET-bound ^{15}N EgtUC based on the WT crystal structure. **l.** Experimental (black) and predicted (blue) R2/R1 values for apo ^{15}N EgtUC based on the AlphaFold2 model. Source data for panels a-b and g-k are available as a Source Data file. Source data for panels c and f are available from the BMRB under accession codes 51423 [https://dx.doi.org/10.13018/BMR51423] (apo-*Sp*EgtUC) and 51424 [https://dx.doi.org/10.13018/BMR51424] (ET-bound *Sp*EgtUC).

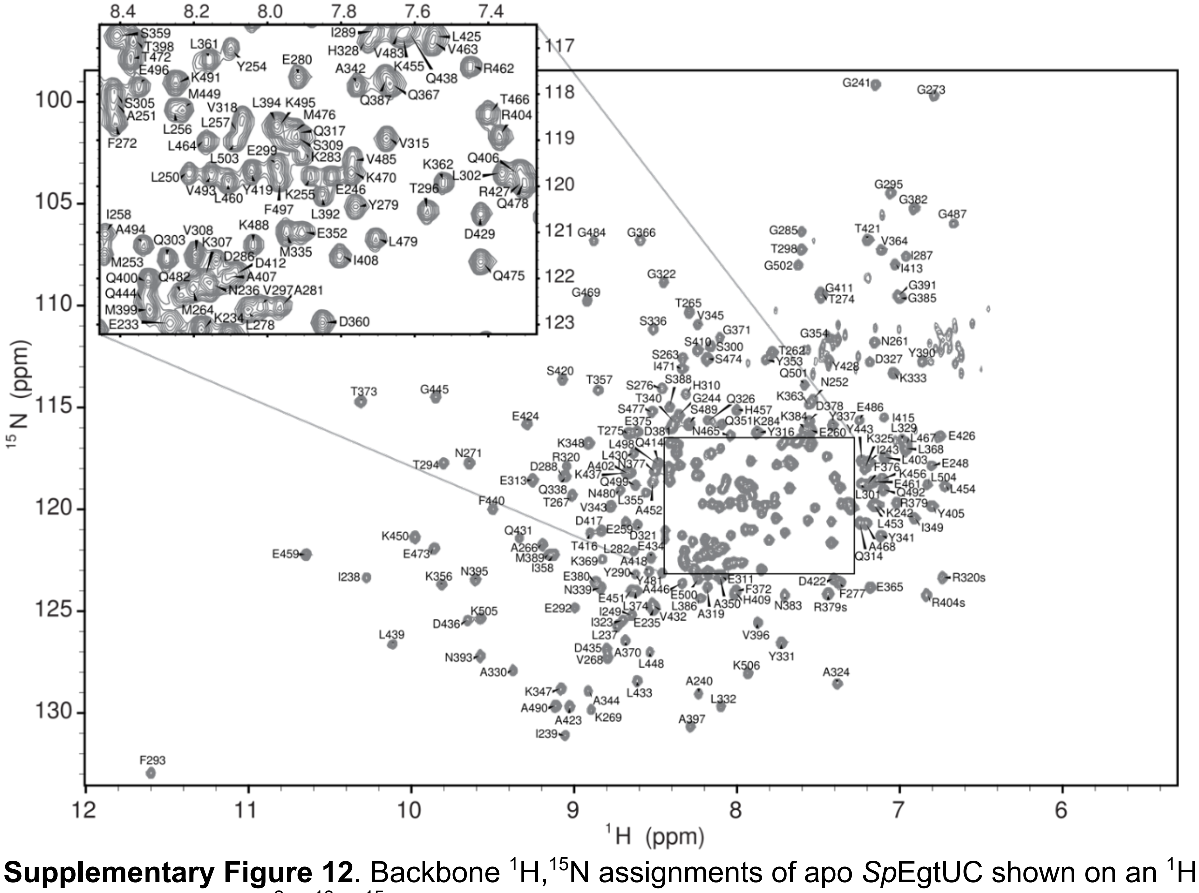

Supplementary Figure 12. Backbone ^{1}H,^{15}N assignments of apo *Sp*EgtUC shown on an ^{1}H,^{15}N TROSY spectrum of ^{2}H,^{13}C,^{15}N-labeled *Sp*EgtUC.

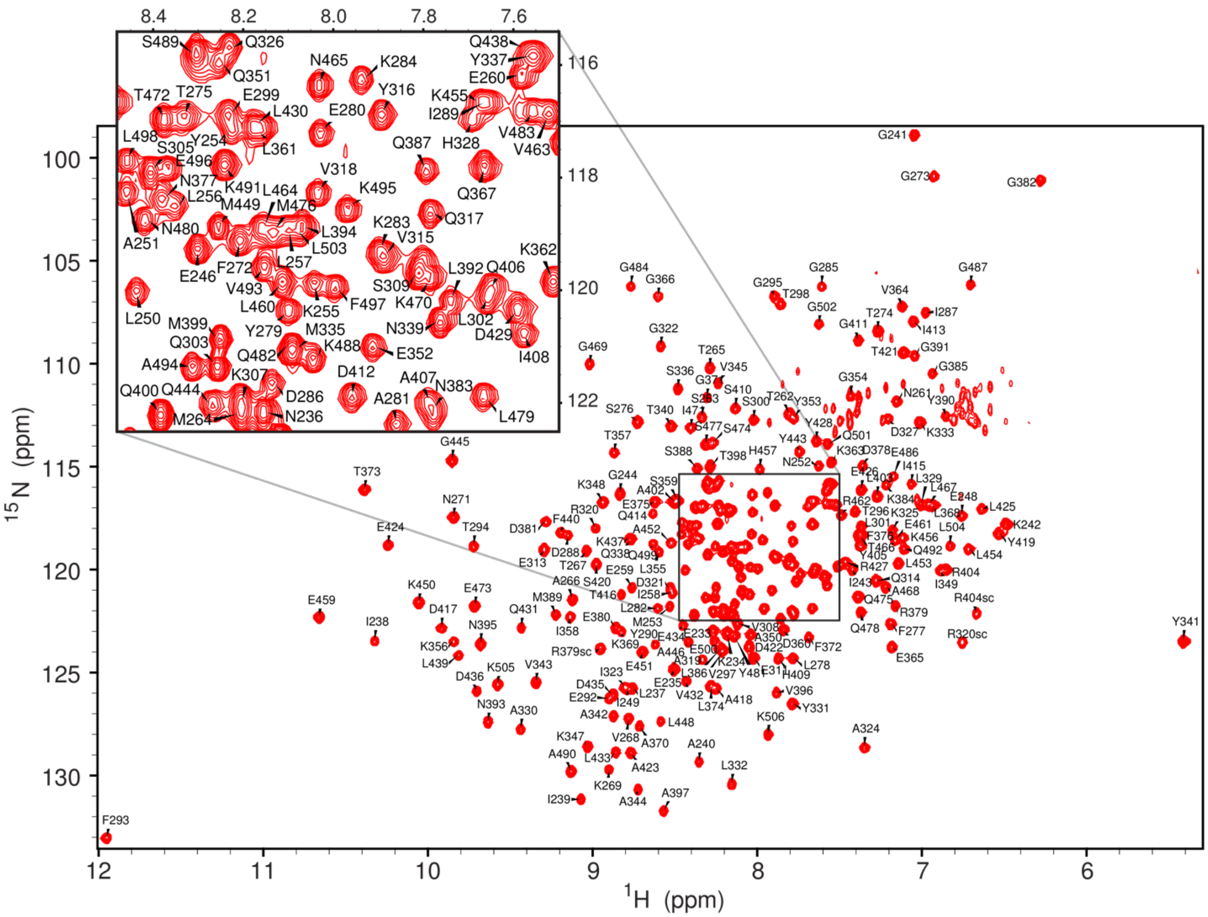

Supplementary Figure 13. Backbone ^{1}H,^{15}N assignments of ET-bound *Sp*EgtUC shown on an ^{1}H,^{15}N TROSY spectrum of ^{2}H,^{13}C,^{15}N-labeled *Sp*EgtUC. Assignments are missing for H310 and V385, which are broadened beyond detection.

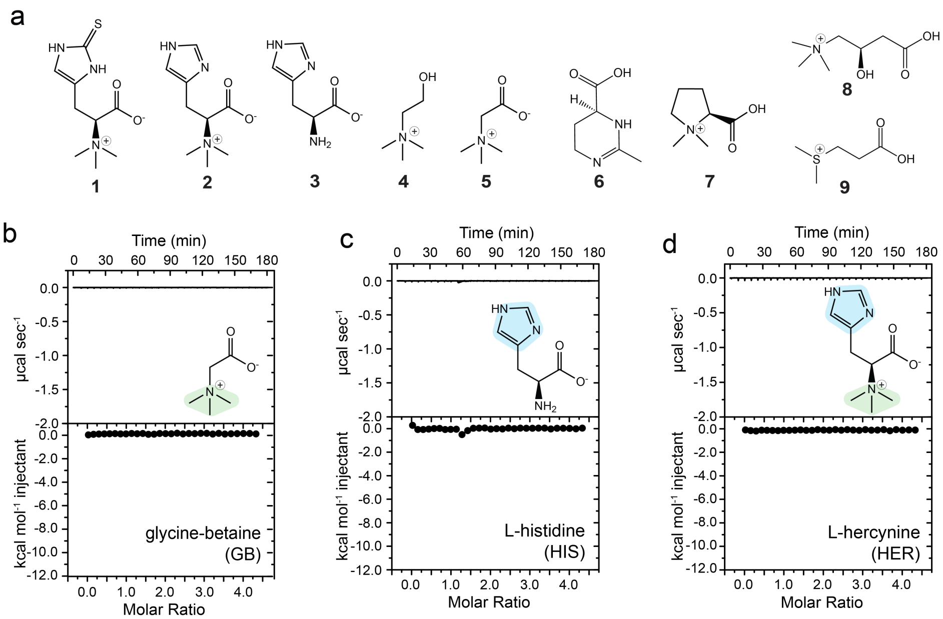

Supplementary Figure 14. Chemical structures of QAC ligands used in this work and representative ITC titrations of wild-type *Sp*EgtUC with non-cognate ligands. **a**, QAC Ligands used in this work. **1,** *L*-ergothioneine (ET); **2,** *L*-hercynine (HER); **3,** *L*-histidine (HIS); **4,** choline (CHO); **5,** glycine-betaine (GB); **6,** ectoine (ECT); **7,** proline-betaine (PB); **8,** *L*-carnitine (CAR); **9,** dimethypropiothetin hydrochloride (DMSP). **b-d**, Isothermal titration calorimetry of the wild-type *Sp*EgtUC titrated with glycine-betaine (**b**), *L*-histidine (**c)** and *L*-hercynine (**d**) under the same conditions as described in the main text for ET titrations (Fig. 3). Source data for panels b-d are available as a Source Data file.

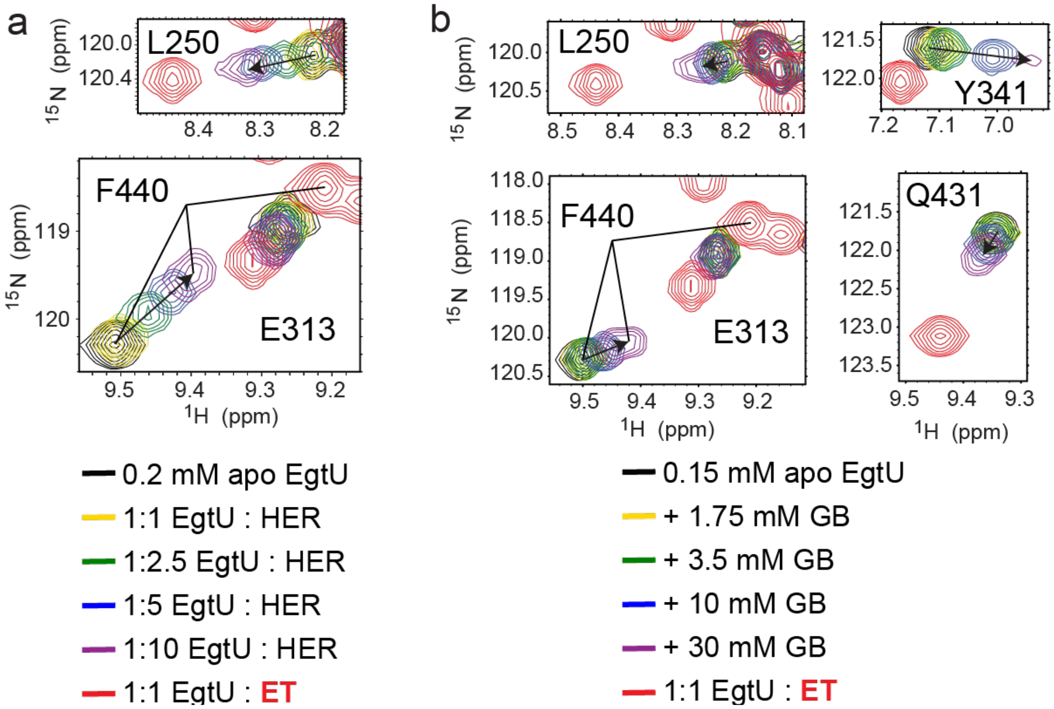

Supplementary Figure 15. NMR-monitored titrations of *Sp*EgtUC with non-cognate and weakly binding ligands *L*-hercynine and glycine betaine. **a**, Movement of the indicated backbone NH crosspeak from the apo-state (black) as *L*-hercynine (HER) is added (yellow to purple), compared to the crosspeak position of ET-bound EgtUC (red) shown for reference. **b**, Movement of the indicated backbone NH crosspeak from the apo-state (black) as glycine-betaine (GB) is added (yellow to purple), compared to the crosspeak position of ET-bound EgtUC (red) shown for reference.

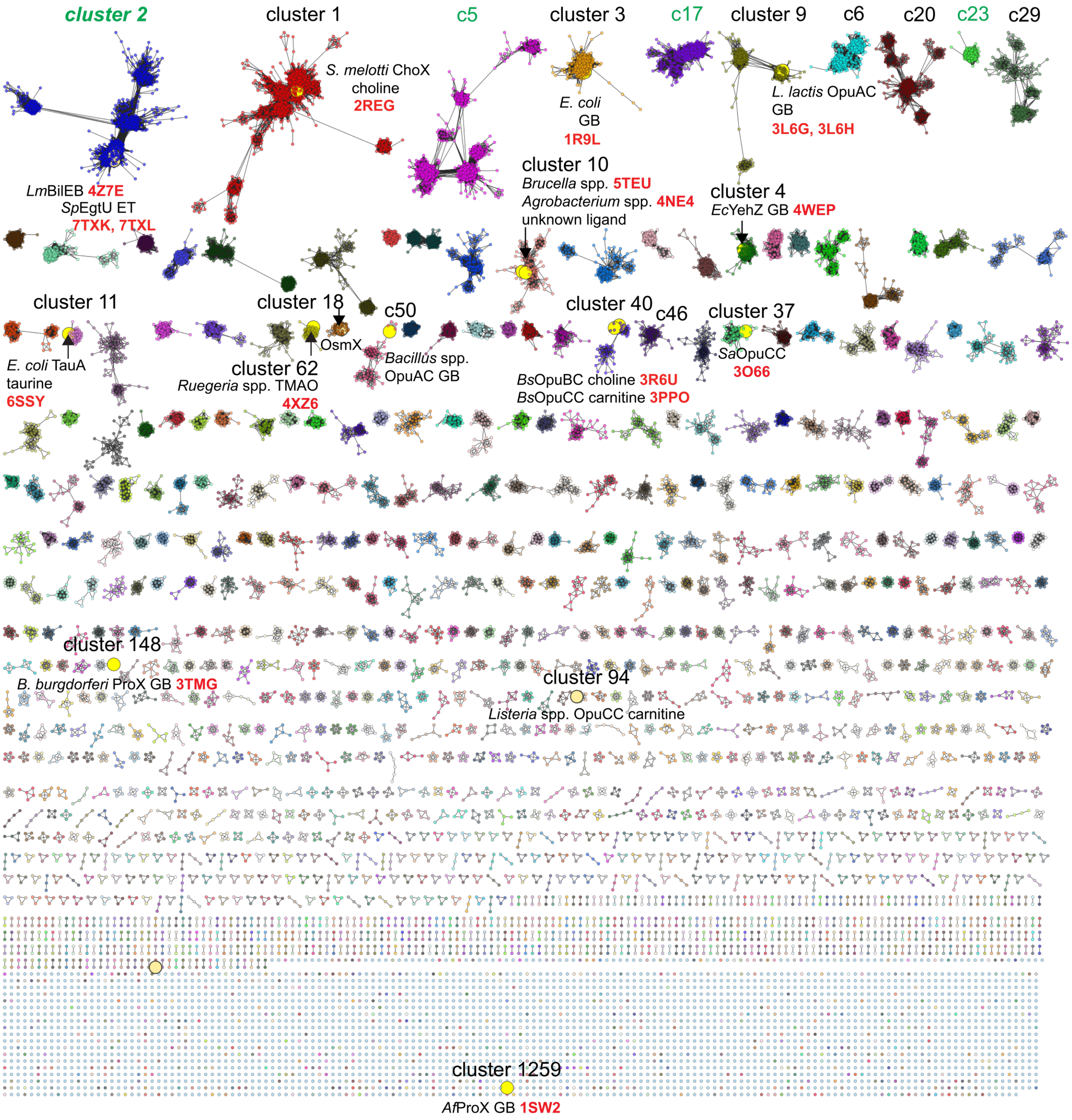

Supplementary Figure 16. **Sequence similarity network (SSN) of solute binding proteins (or domains) most closely related to *Sp*EgtUC.** Full sequence similarity network (SSN) analysis[5] of the osmoprotectant subclass of solute binding proteins (or domains) (SBPs/SPDs) resulting in 2,044 clusters and the 2458 singletons. Clusters are ranked numerically from that containing the largest (cluster 1; c1) to the smallest number of sequences and are arranged from *upper left* to *lower right* on the basis of metanode cluster count. Those sequences for which SwissProt annotations exist are highlighted (*yellow circle*), along with their trivial names, known or inferred ligand specificity and the organism (*Sp*, *S. pneumoniae*; *Lm*, *Listeria monocytogenes*, *Bs*, *B. subtilis*; *Sa*, *Staphylococcus aureus*, *Ec*, *Escherichia coli*, *Af*, *Archaeoglobis fulgidus*). Those sequences for which crystal structures are available are highlighted with a Protein

Databank (PDB) accession number (in *red*); if a ligand is known, it is also indicated. The subject of this work are the sequences associated with SSN cluster 2 with *Sp*EgtUC structures reported here also shown (7TXL, 7TXK). GB, glycine betaine; ET, ergothioneine. Source data available as a Source Data file.

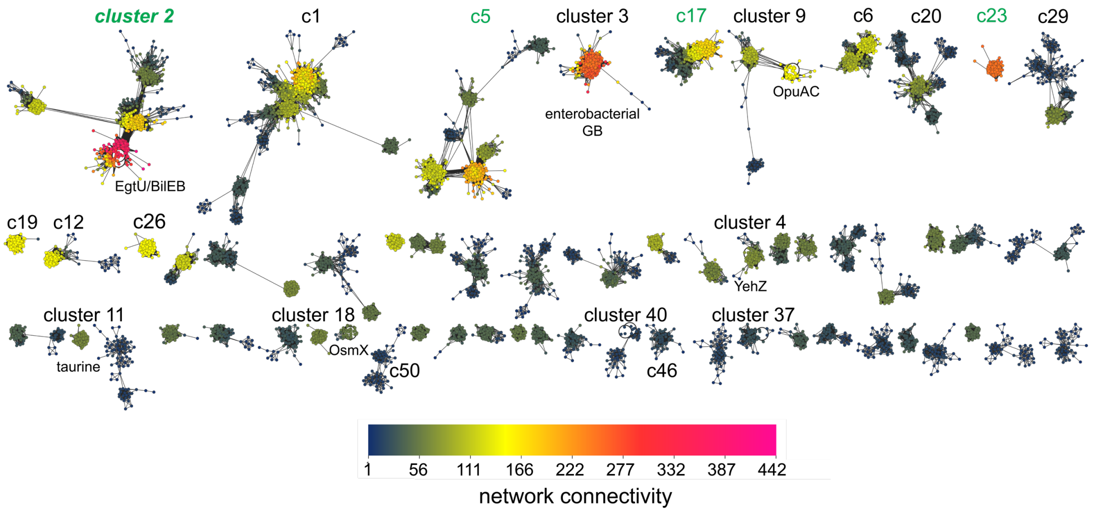

Supplementary Figure 17. Network connectivity analysis. Network connectivity representation for the top 58 SSN clusters (based on metanode cluster count). All clusters below this threshold are characterized by network connectivity values of ≤40 (see scale bar, *bottom*). The EgtU/BilEB subcluster in SSN cluster 2 (*lower*) along with SSN cluster 3 sequences encoding curated as enterobacterial (Gram-negative) glycine betaine/proline betaine SBPs (GB), are among the most highly similar groups of sequences in the SSN database.

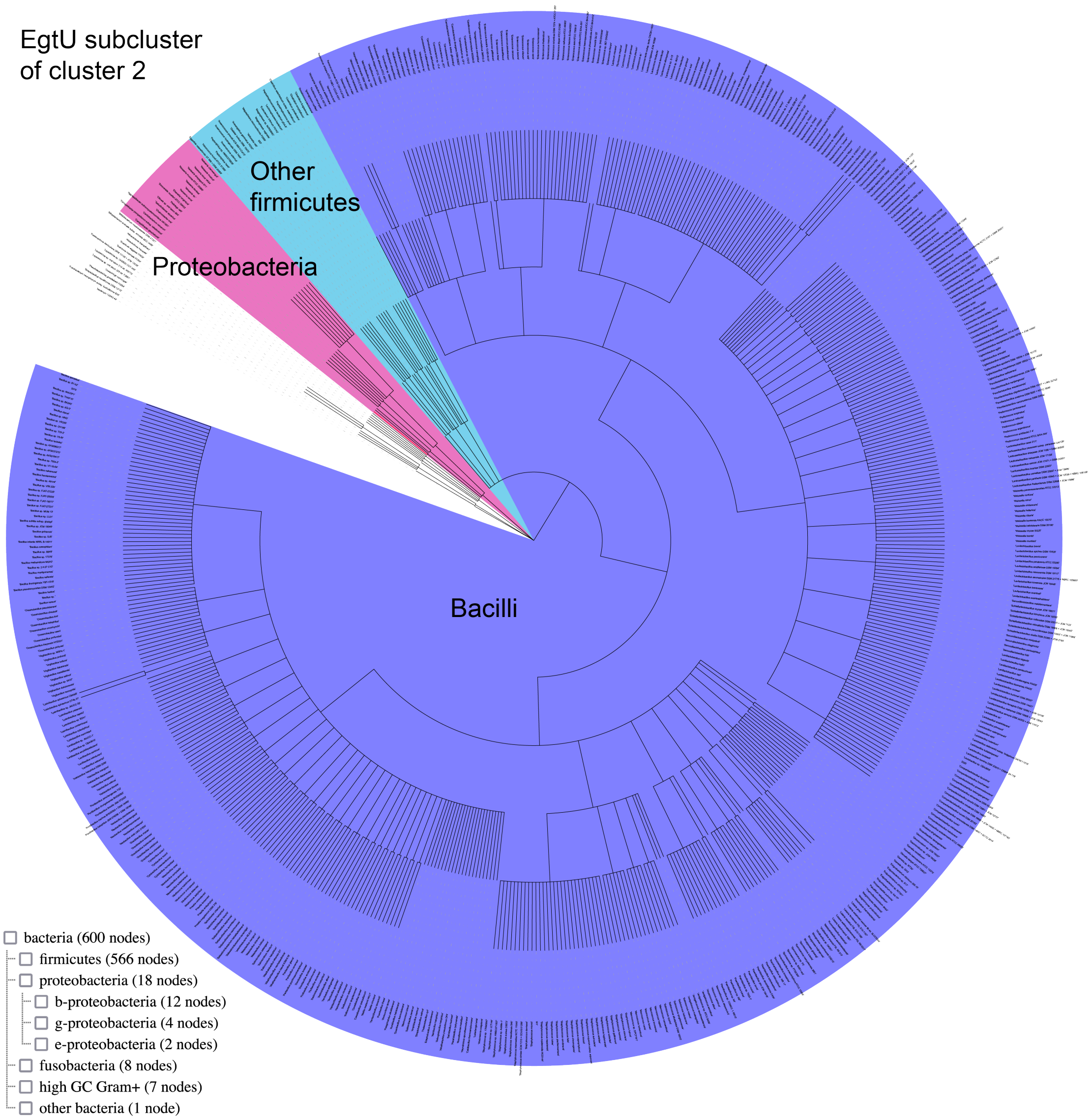

Supplementary Figure 18. The biological range determined for the EgtU subcluster of cluster 2. Taxonomy IDs extracted from the SSN analysis were supplied to the server NCBI Taxonomy common tree (https://www.ncbi.nlm.nih.gov/Taxonomy/CommonTree/wwwcmt.cgi)[6] to generate the phylogenetic tree, which was then visualized using iTOL.

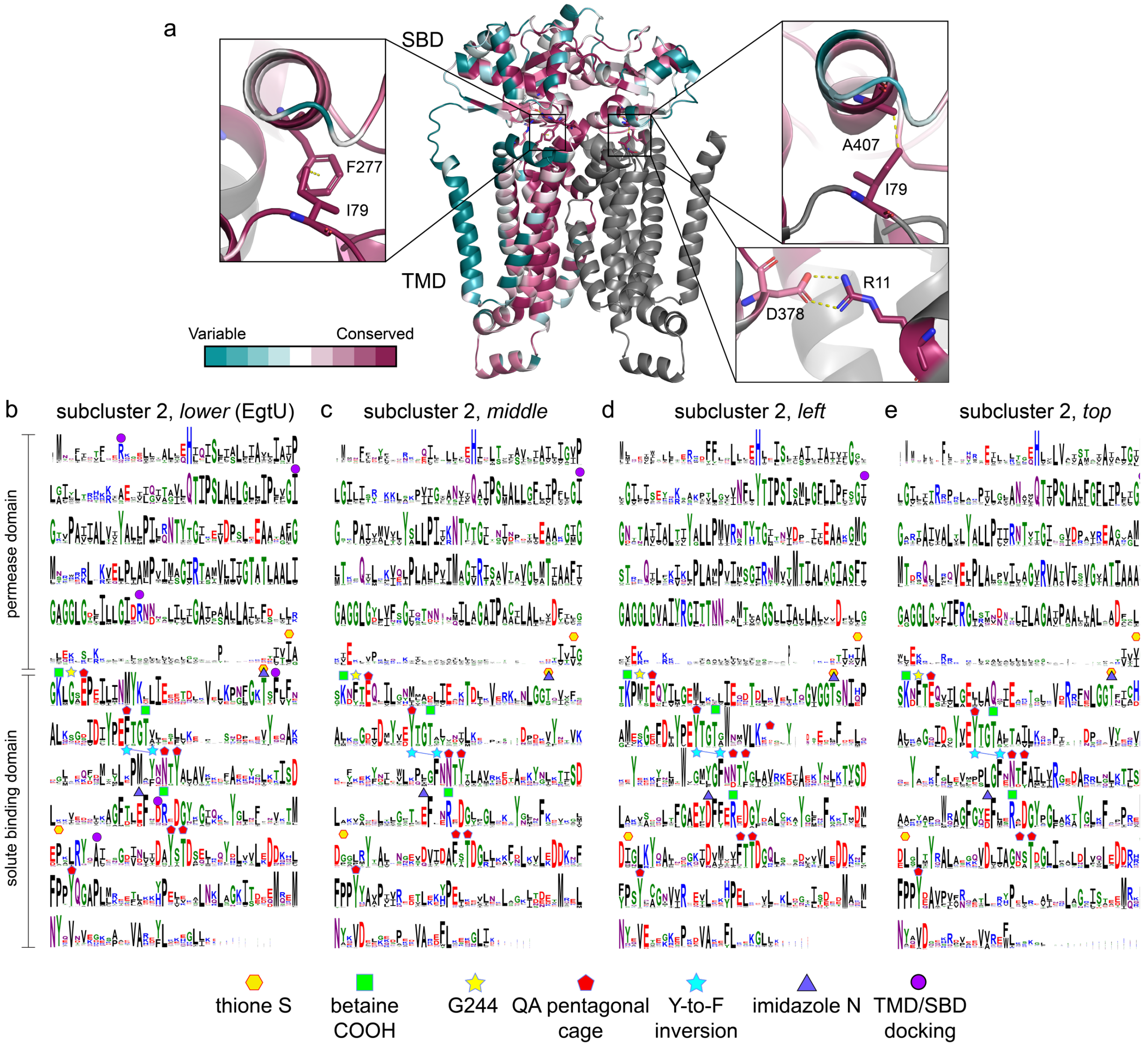

Supplementary Figure 19. Sequence conservation maps of SSN cluster 2 subclusters. **a,** sequence conservation[7] mapped onto an AlphaFold2 model of *Sp*EgtUBC with the SBD docked onto the TMDs. Conserved interface residues are highlighted. **b-e**, Subclusters *lower* (*Sp*EgtU-like) (**b**), *middle* (*C. difficile* sequences)[8] (**c**), *left* (Streptococci, Clostridia, *H. pylori*) (**d**) and *upper* (Acidobacteria, Cyanobacteria) (**e**) multiple sequence alignments displayed as sequence logos[9]. Symbols (shown for reference) indicate conserved features of the ET binding site identified in the *Sp*EgtU subcluster of SSN cluster 2 (panel **b**) (see also Fig. 6c, main text), many of which are not broadly conserved in the other SSN cluster 2 subclusters (panels **c**-**e**). This analysis suggests that these other subclusters possess a QAC ligand specificity profile that is distinct from that of EgtU.

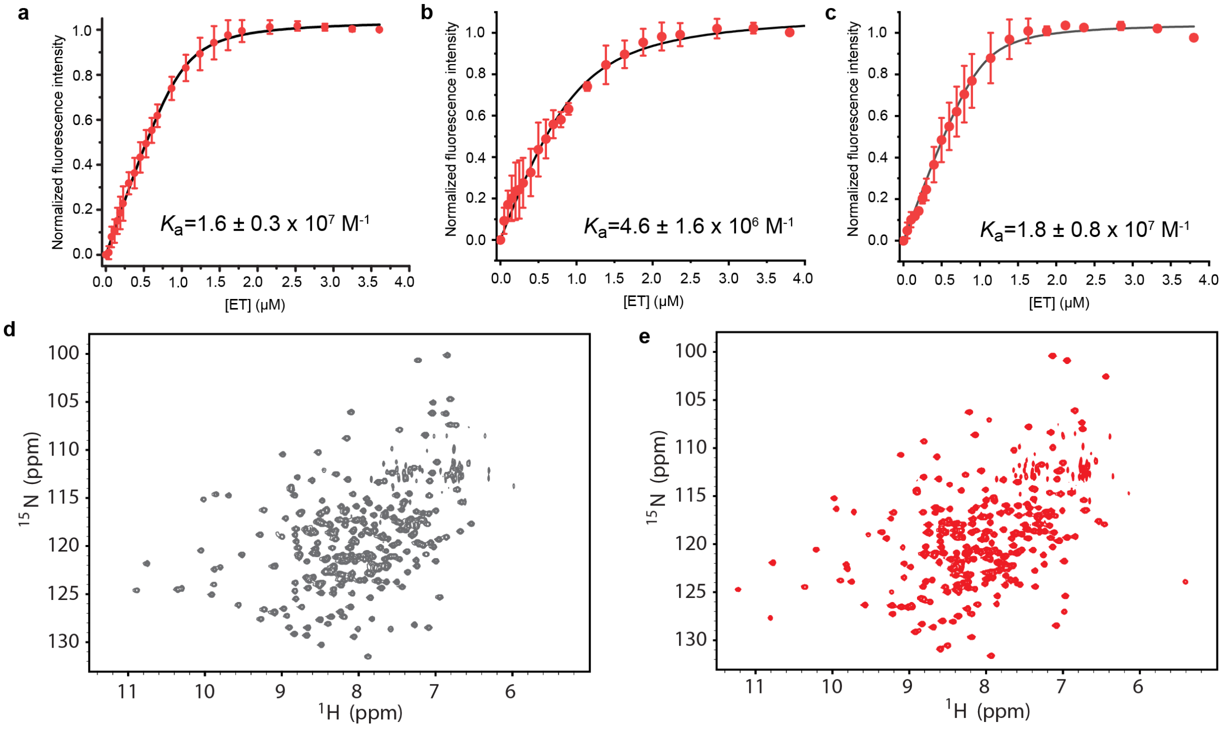

Supplementary Figure 20. ET-binding properties of candidate EgtU homologs from other firmicutes. a-c, Tyrosine fluorescence titrations of 1.0 µM *Ef*EgtUC (**a**), 1.0 µM *Sa*EgtUC (**b**) and 1.0 µM *Lm*EgtUC (**c**) with ET. The continuous line through the data shows the results of a fit to a 1:1 binding model with the K_a indicated using DynaFit[10] with the parameters compiled in Table 1, main text. Each data point is shown as the mean and standard deviation of three (*Ef*EgtUC and *Sa*EgtUC) or two (*Lm*EgtUC) independent replicates. **d,** ^{1}H,^{15}N TROSY spectrum of *Ef*EgtUC. **e,** ^{1}H,^{15}N TROSY spectrum of *Ef*EgtUC in complex with equimolar ET. Source data for panels a-c are available as a Source Data file.

SUPPLEMENTARY TABLES

Supplementary Table 1. Data collection and refinement statistics

	*Sp*EgtUC$_{CTT}$	*Sp*EgtUC
Data collection		
Wavelength (Å)	1.07216	1.07216
Space group	F222	F222
Cell dimensions		
a, b, c (Å)	118.00 129.02 206.91	119.87 128.74 207.54
a, b, g (°)	90.00 90.00 90.00	90.00 90.00 90.00
Resolution * (Å)	43.54 – 1.78 (1.82 – 1.78)	43.86 – 2.44 (2.54 – 2.44)
R_{sym}	0.081 (0.983)	0.167 (1.086)
R_{meas}	0.087 (1.062)	0.195 (1.282)
R_{pim}	0.033 (0.400)	0.073 (0.496)
Total reflections	524963 (29990)	211621 (22388)
No. unique reflections	75117 (4251)	29967 (3349)
CC1/2	0.998 (0.824)	0.994 (0.714)
I/s(I)	13.7 (1.6)	8.5 (1.6)
Completeness (%)	99.9 (100.0)	99.9 (99.9)
Multiplicity	7.0 (7.1)	7.1 (6.7)
Wilson B-factor	25.70	38.59
Refinement		
Resolution (Å)	43.54 – 1.78	40.4 – 2.44
No. unique reflections	75056 (7462)	29938 (2942)
R_{work}	0.1586 (0.2357)	0.1834 (0.2696)
R_{free}	0.1863 (0.2671)	0.2402 (0.3417)
R.m.s.d values		
Bond lengths (Å)	0.007	0.004
Bond angles (°)	0.96	0.603
No. atoms		
Protein	4256	4324
Ligand/ions	115	70
solvent	539	156
B-factors (Å^2)		
Protein	34.67	48.79

Ligand/ions	47.85	42.07
solvent	40.92	41.49
Ramachandran plot		
Favored (%)	98.0	98.0
Allowed (%)	1.6	1.6
Outliers (%)	0.4	0.4
Clashscore	3.25	7.21
Rotamer outliers (%)	0.2	1.3
PDB code	7TXL	7TXK

*Highest-resolution shell values are shown in parentheses.

Supplementary Table 2. List of candidate C-H•••S hydrogen bonding interactions in the EgtUC-ET complex.[a]

Candidate C-H•••S H-bond donor	Distance, H•••S (Å)	Angle, C-H•••S (º)
K242 HB3	2.8	177
I243 HB	3.0	126
T274 HA	3.1	152
T274 HG22	3.0	152

[a]See Supplementary Figure 9c and Fig. 2, main text, for structural representations of these C-H•••S hydrogen bonds.

Supplementary Table 3. List of water molecules and associated B-factors in the structures of *Sp*EgtUC$_{CTT}$ and *Sp*EgtUC[a]

*Sp*EgtUC$_{CTT}$				*Sp*EgtUC			
Ori ID#	New ID#	Occupancy	B factor	Ori ID#	New ID#	Occupancy	B factor
HOH 23	W1	1	24.73	HOH 5	W1	1	31.20
HOH 22	W2	1	24.32	HOH 8	W2	1	24.97
HOH 33	W3	1	23.90	N/A	W3	N/A	N/A
HOH 20	W4	1	23.92	HOH 49	W4	1	31.52
HOH 10	W5	1	22.78	HOH 32	W5	1	27.79
HOH 11	W6	1	22.51	HOH 60	W6	1	29.67
HOH 32	W7	1	23.24	HOH 43	W7	1	34.67
HOH 6	W8	1	21.51	HOH 4	W8	1	38.00
HOH 25	W9	1	22.03	HOH 51	W9	1	34.67
HOH 35		1	26.39	HOH 68		1	41.48
HOH 59		1	24.77	HOH 133		1	38.62
HOH 110		1	30.93	HOH 79		1	41.05
HOH 317		1	42.01	HOH 116		1	49.28
HOH 66		1	25.63	HOH 104		1	40.73
HOH 356		1	35.41	HOH 96		1	35.69
HOH 183		1	36.34				
HOH 255		1	40.86				
HOH 79		1	29.09				
HOH 102		1	29.60				
HOH 58		1	26.19				
HOH 5		1	22.78				

[a]From PDB entries 7TXL and 7TXK, respectively. Water molecules 1-9 (W1-W9) correspond to those shown in Fig. 4, main text.

Supplementary Table 4. List of *Streptococcus pneumoniae* D39 strains in this work

Strain ID	Genotype	Ref
IU1781	D39 *rpsL1* (WT)	[11]
IU18405	D39 *rpsL1* Δ*spd_1642,* markerless (Δ*egtU*^{ML})	This work
IU18789	D39 *rpsL1* Δ*spd_1642,* markerless, repaired (Δ*egtU*^{ML} repaired)	This work
IU19171	D39 *rpsL1 spd_1643,* K40A (*egtUA*^{K40A})	This work
IU19173	D39 *rpsL1 spd_1643,* E161Q (*egtUA*^{E161Q})	This work
IU19175	D39 *rpsL1* Δ*spd_1643* WT (Δ*egtUA*^{reparied})	This work

Supplementary Table 5. Primers used in this study[a]

Primer IDs	Sequence 5' to 3'
***Sp*EgtU mutant strains**	
1642_outside_FP	GGAACTGCGACTTCTGCGC
1642_outside_RP	CTCCCAGACTGTTTCACTCCCG
1642_KanrpsL_NT_RP	CATTATCCATTAAAAATCAAACGGATCCTAATCCTGAAAAGTTGCAATTAAATTAGTC
1642_KanrpsL_CT_FP	CAAAAGCATAAGGAAAGGGGCCCCTCCAAGAACAAGGTTTGTTGAAGAAATGATGG
1642_ML_Rev	CCTTGTTCTTGGAGATCCTGAAAAGTTGCAATTAAATTAGTCATGAATACTACC
1642_ML_Fwd	GCAACTTTTCAGGATCTCCAAGAACAAGGTTTGTTGAAGAAATGATG
1643_KanrpsL_NT_FP	CGGAGTTAGGCAAGTCCCAGAAAG
1643_KanrpsL_NT_RP	CATTATCCATTAAAAATCAAACGGATCCTAGCGCAGTGCTACATTTTTGTATTC
1643_KanrpsL_CT_FP	CAAAAGCATAAGGAAAGGGGCCCGTTGCAGACTTGTTTGGAGGTAGTATTCAT
1643_KanrpsL_CT_RP	GATAAACCTGCTCTGGCTCATGACTC
1643_K40A_CT_FP	GGGTCAGGTGCGACGACCATGCTCAAGATGATTAACCG
1643_K40A_NT_RP	GCATGGTCGTCGCACCTGACCCAGAAGGCCC
1643_E161Q_CT_FP	CTCCTCATGGATCAACCCTTTTCAGCCTTGGATGC
1643_E161Q_NT_RP	CTGAAAAGGGTTGATCCATGAGGAGAATCTTGGGC
**Protein expression**	
*Sp*EgtU-SBD_pSUMO_FP	GAACAGATTGGATCCCATATGGAGAAGGAAAACTTGATTATTGCTGGGAAAATAGGC
*Sp*EgtU-SBD_pSUMO_RP	GTGCTCGAGTGCGGCCGCAAGCTTTCATTTCTTCAACAAACCTTGTTCTTGGAGAAACTC
*Sp*EgtU-SBD_Y419F_FP	GGGGATATTCAAATCACGGATGCCTTTTCGACTGATGCGGAATTGGAGC
*Sp*EgtU-SBD_Y419F_RP	GCTCCAATTCCGCATCAGTCGAAAAGGCATCCGTGATTTGAATATCCCC
*Sp*EgtU-SBD_G244F_FP	GGAAAACTTGATTATTGCTGGGAAAATATTCCCAGAACCAGAAATTTTGGCTAATATG
*Sp*EgtU-SBD_G244F_RP	CATATTAGCCAAAATTTCTGGTTCTGGGAATATTTTCCCAGCAATAATCAAGTTTTCC
*Sp*EgtU-SBD_F277W_FP	GTTAAACCGAATTTTGGGACGACAAGTTGGCTTTATGAAGCTCTGAAAAAAGGTG

*Sp*EgtU-SBD_F277W_RP	CACCTTTTTTCAGAGCTTCATAAAGCCAACTTGTCGTCCCAAAATTCGGTTTAAC
*Sp*EgtU-SBD_L374C_FP	GCAGCTGAAGGCAGGCTTTACGTGTGAGTTTAACGACCGTGAAGATGGAAATAAGG
*Sp*EgtU-SBD_L374C_RP	CCTTATTTCCATCTTCACGGTCGTTAAACTCACACGTAAAGCCTGCCTTCAGCTGC
*Sp*EgtU-SBD_F293Y_FP	GAAAAAAGGTGATATTGACATTTATCCTGAATATACCGGTACGGTGACTGAAAG
*Sp*EgtU-SBD_F293Y_RP	CTTTCAGTCACCGTACCGGTATATTCAGGATAAATGTCAATATCACCTTTTTTC
*Sp*EgtU-SBD_E375Q_FP	CTGAAGGCAGGCTTTACGCTTCAGTTTAACGACCGTGAAGATGGAAATAAG
*Sp*EgtU-SBD_E375Q_RP	CTTATTTCCATCTTCACGGTCGTTAAACTGAAGCGTAAAGCCTGCCTTCAG
SpEgtU-SBD_CTT_RP	GTGCGGCCGCAAGCTTTCATTTCTTCAACAAACCTGTTCTTGGAGAAACTCCTTGGCTA
*Ef*EgtU-SBD_pSUMO_FP	GAACAGATTGGAGGATCCCATATGAAGGAAAAACAGCTGACAATTGCTGGC
*Ef*EgtU-SBD_pSUMO_RP	TCGAGTGCGGCCGCAAGCTTTTATTTAAGAAGATTCTTTTCTTTTAAATAGTCTTTCGCC
*Sp*EgtU-S_P1	TGGTTCTGGTGATGGTTGAAGCAAACTTTCAGTCACCG
*Sp*EgtU-S_P2	GAATACAACTTTCCTCCTCCCAAGGTGAGTCATGAGCCAG
*Sp*EgtU-S_P3	GCTTCAACCATCACCAGAACCAAACGTATACATCACTGCTGACAAACAAAAAAACGG
*Sp*EgtU-S_P4	CATACTACCACCTGTACCACCCTTATAAAGCTCATCCATGCCGTGAGTGATACC
*Sp*EgtU-S_P5	GGGTGGTACAGGTGGTAGTATGAAAGGAGAAGAGCTGTTCACAGGTG
*Sp*EgtU-S_P6	CACCTTGGGAGGAGGAAAGTTGTATTCAAGTTTGTGTCCAAGGATGTTTCC
*Sa*EgtU-SBD_pSUMO_FP	GAACAGATTGGAGGATCCCATATGGGTGATAAAATTACGTTAGCTGGAAAGCTTGG
*Sa*EgtU-SBD_pSUMO_RP	GCTCGAGTGCGGCCGCAAGCTTTTATTTGATTAACCCTTTTGCTTTTAAATAATCTTTCG
*Lm*EgtU-SBD_pSUMO_FP	AGATTGGAGGATCCCATATGTCGGATAAAAAGGAAATTACAATTGCTGGTAAATTAGG
*Lm*EgtU-SBD_pSUMO_RP	CTCGAGTGCGGCCGCAAGCTTTATTTAATAATACCTTGATCTTTCAAATAGTCTTTGGC

[a]All sequences are written 5’ to 3’. FP, forward primer; RP, reverse primer.

Supplementary Table 6. Molar extinction coefficients of purified proteins at 280 nm (ε_{280}) used in this work

Protein	Mutation	MW, Da	molar extinction coefficient (e_{280})[a], M^{-1} cm^{-1}
*Sp*EgtUC	WT	31032.4	20,860
	CTT[b]	30695.0	20,860
	Y419F	31016.4	19,370
	G244F	31122.5	20,860
	F293Y	31048.4	22,350
	E375Q	31031.4	20,860
	F227W/L374C	31061.4	26,360
*Sp*EgtUC-GFP	–	58508.4	41,260
*Ef*EgtUC	WT	31373.7	22,350
*Sa*EgtUC	WT	31638.6	22,350
*Lm*EgtUC[c]	WT	31351.8	23,840

[a]Assuming that all Cys residues are in the reduced form. [b]CTT, C-terminally truncated. [c]Also known as BilEB[12].

SUPPLEMENTARY REFERENCES

1. Jacobsen, F.E., Kazmierczak, K.M., Lisher, J.P., Winkler, M.E. & Giedroc, D.P. Interplay between manganese and zinc homeostasis in the human pathogen *Streptococcus pneumoniae. Metallomics* **3**, 38-41 (2011).
2. Ramos-Montanez, S., Kazmierczak, K.M., Hentchel, K.L. & Winkler, M.E. Instability of ackA (acetate kinase) mutations and their effects on acetyl phosphate and ATP amounts in *Streptococcus pneumoniae* D39. *J Bacteriol* **192**, 6390-400 (2010).
3. Krogh, A., Larsson, B., von Heijne, G. & Sonnhammer, E.L. Predicting transmembrane protein topology with a hidden Markov model: Application to complete genomes. *J Mol Biol* **305**, 567-80 (2001).
4. Omasits, U., Ahrens, C.H., Muller, S. & Wollscheid, B. Protter: Interactive protein feature visualization and integration with experimental proteomic data. *Bioinformatics* **30**, 884-6 (2014).
5. Zallot, R., Oberg, N. & Gerlt, J.A. The EFI web resource for genomic enzymology tools: leveraging protein, genome, and metagenome databases to discover novel enzymes and metabolic pathways. *Biochemistry* **58**, 4169-4182 (2019).
6. Letunic, I. & Bork, P. Interactive Tree Of Life (iTOL) v5: An online tool for phylogenetic tree display and annotation. *Nucleic Acids Res* **49**, W293-W296 (2021).
7. Ashkenazy, H. et al. ConSurf 2016: An improved methodology to estimate and visualize evolutionary conservation in macromolecules. *Nucleic Acids Res* **44**, W344-50 (2016).
8. Michel, A.M. et al. Cellular adaptation of *Clostridioides difficile t*o high salinity encompasses a compatible solute-responsive change in cell morphology. *Environ Microbiol* **24**, 1499-1517 (2022).
9. Crooks, G.E., Hon, G., Chandonia, J.M. & Brenner, S.E. WebLogo: A sequence logo generator. *Genome Res* **14**, 1188-90 (2004).
10. Kuzmic, P. Program DYNAFIT for the analysis of enzyme kinetic data: Application to HIV proteinase. *Anal Biochem* **237**, 260-273 (1996).
11. Lanie, J.A. et al. Genome sequence of Avery's virulent serotype 2 strain D39 of *Streptococcus pneumoniae* and comparison with that of unencapsulated laboratory strain R6. *J Bacteriol* **189**, 38-51 (2007).

12. Ruiz, S.J., Schuurman-Wolters, G.K. & Poolman, B. Crystal structure of the substrate-binding domain from *Listeria monocytogenes* bile-resistance determinant BilE. *Crystals* **6**, 162 (2016).

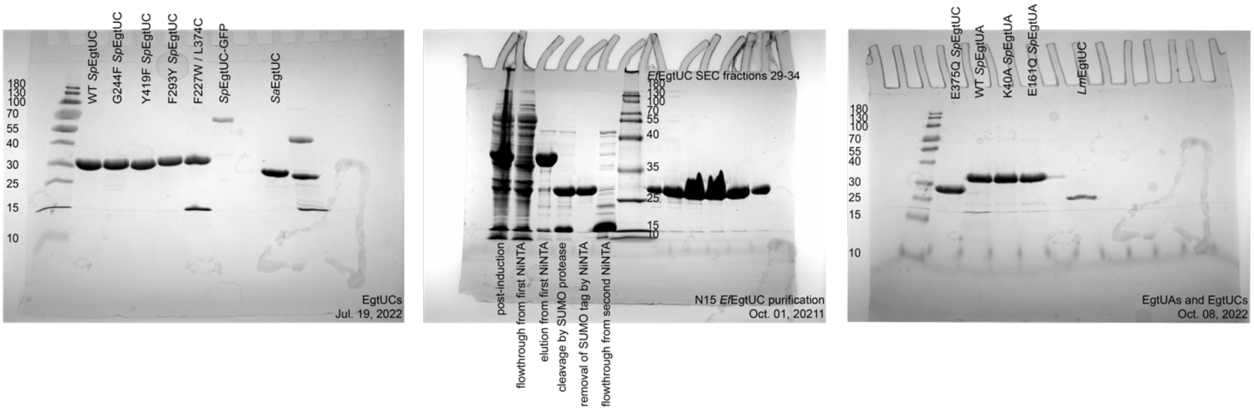

Uncropped SDS-PAGE gel images for Supplementary Figure 7f. Full gels and the dates on which each of these gels were run is shown.
