# Supplementary material for: Discovery and structure of a widespread bacterial ABC transporter specific for ergothioneine
Source: Nat Commun. 2022 Dec 8;13:7586. doi: 10.1038/s41467-022-35277-3 (PMC9732360; doi:10.1038/s41467-022-35277-3)
Supplement: Supplementary file 2 — Reporting Summary [file 41467_2022_35277_MOESM2_ESM.pdf]

nature portfolio

Corresponding author(s): Giedroc, David P.

Last updated by author(s): Nov 17, 2022

Reporting Summary

Nature Portfolio wishes to improve the reproducibility of the work that we publish. This form provides structure for consistency and transparency in reporting. For further information on Nature Portfolio policies, see our Editorial Policies and the Editorial Policy Checklist.

Statistics

For all statistical analyses, confirm that the following items are present in the figure legend, table legend, main text, or Methods section.

n/a	Confirmed	
☐	☒	The exact sample size (*n*) for each experimental group/condition, given as a discrete number and unit of measurement
☐	☒	A statement on whether measurements were taken from distinct samples or whether the same sample was measured repeatedly
☐	☒	The statistical test(s) used AND whether they are one- or two-sided<br>*Only common tests should be described solely by name; describe more complex techniques in the Methods section.*
☒	☐	A description of all covariates tested
☒	☐	A description of any assumptions or corrections, such as tests of normality and adjustment for multiple comparisons
☐	☒	A full description of the statistical parameters including central tendency (e.g. means) or other basic estimates (e.g. regression coefficient) AND variation (e.g. standard deviation) or associated estimates of uncertainty (e.g. confidence intervals)
☒	☐	For null hypothesis testing, the test statistic (e.g. *F*, *t*, *r*) with confidence intervals, effect sizes, degrees of freedom and *P* value noted<br>*Give P values as exact values whenever suitable.*
☒	☐	For Bayesian analysis, information on the choice of priors and Markov chain Monte Carlo settings
☒	☐	For hierarchical and complex designs, identification of the appropriate level for tests and full reporting of outcomes
☒	☐	Estimates of effect sizes (e.g. Cohen's *d*, Pearson's *r*), indicating how they were calculated

Our web collection on statistics for biologists contains articles on many of the points above.

Software and code

Policy information about availability of computer code

Data collection	Protein Sequence similarity network (SSN) collection is performed by the Enzyme Similarity Tool (EFI-EST), database UniProt 2022_01 and InterPro 88. Predicted protein structure model is generated by using AlphaFold v2.1.0 Colab version. More details are provided in the Methods section.
Data analysis	Protein sequence similarity network (SSN) data analysis and organized in Cytoscape v3.9.1. NMR data analysis with CARA v1.9.1, Sparky v3.115 and HYDRONMR v7c. Protein crystallography data analysis with PHENIX v1.15, MolProbity v4.5 and Pymol v3.3. Fluorescence based titration data were fitted with Dynafit v4. ITC data analysis with the MicroCal ITC data analysis tool in Origin. Mass spectrometry data analysis using Waters MassLynx v4.2 software. More details are provided in the Methods section.

For manuscripts utilizing custom algorithms or software that are central to the research but not yet described in published literature, software must be made available to editors and reviewers. We strongly encourage code deposition in a community repository (e.g. GitHub). See the Nature Portfolio guidelines for submitting code & software for further information.

Data

Policy information about availability of data

All manuscripts must include a data availability statement. This statement should provide the following information, where applicable:

- Accession codes, unique identifiers, or web links for publicly available datasets
- A description of any restrictions on data availability
- For clinical datasets or third party data, please ensure that the statement adheres to our policy

All primary data are provided as a Source File. The crystallographic structures have been deposited in the Protein Data Bank under accession codes 7TXL and 7TXK. NMR data are available from the BMRB under accession codes 51423 and 51424 for the apo and ET-bound states of the EgtU SBD, respectively. The model of the

apo EgtU SBD is available in ModelArchive at https://modelarchive.org/doi/10.5452/ma-xwg27.

Field-specific reporting

Please select the one below that is the best fit for your research. If you are not sure, read the appropriate sections before making your selection.

☒ Life sciences ☐ Behavioural & social sciences ☐ Ecological, evolutionary & environmental sciences

For a reference copy of the document with all sections, see nature.com/documents/nr-reporting-summary-flat.pdf

Life sciences study design

All studies must disclose on these points even when the disclosure is negative.

Sample size	No sample size calculation was performed since we present the results of only analytical experiments in this manuscript. For fluorescence-based titrations, the experiments were performed as three independent replicates. For the ITC titrations, the experiments were performed in two or three independent replicates (titrations). For mass spectrometry, the experiments were performed in three independent biological replicates. We judged these sample sizes to be sufficient on the basis of good agreement among replicate trials.
Data exclusions	No systematic data exclusions were applied in this study. All replicates were considered in the data analysis and processing.
Replication	Experiments were replicated in duplicate trials or in three independent trials (the vast majority of experiments were performed in triplicate, with the number of replicates now described in all figure legends). Replication was deemed successful when good agreement was achieved was achieved from separate independent trials.
Randomization	Randomization was not applicable or relevant to this study since we present the aggregate results of independent trials which were not acquired in any ordered way.
Blinding	Blinding was not relevant to the study, because no manual counting or scoring was performed to obtain the data.

Reporting for specific materials, systems and methods

We require information from authors about some types of materials, experimental systems and methods used in many studies. Here, indicate whether each material, system or method listed is relevant to your study. If you are not sure if a list item applies to your research, read the appropriate section before selecting a response.

Materials & experimental systems

n/a	Involved in the study	
☒	☐	Antibodies
☒	☐	Eukaryotic cell lines
☒	☐	Palaeontology and archaeology
☒	☐	Animals and other organisms
☒	☐	Human research participants
☒	☐	Clinical data
☒	☐	Dual use research of concern

Methods

n/a	Involved in the study	
☒	☐	ChIP-seq
☒	☐	Flow cytometry
☒	☐	MRI-based neuroimaging
